# Xanthan Oligosaccharide Seed Coating Promotes Wheat Growth and Stress-Related Physiological Reprogramming via Hormone Signaling and Plant–Microbe Associations

**DOI:** 10.3390/plants15152340

**Published:** 2026-07-29

**Authors:** Miaowen Hao, Chunshu Chen, Jiapeng Li, Lingxin Lv, Hong Jin, Fan Yang

**Affiliations:** School of Biological Engineering, Dalian Polytechnic University, Dalian 116034, China; only_hmw@163.com (M.H.); 15714114068@163.com (C.C.); 19819205955@163.com (J.L.); cccllx2021@163.com (L.L.)

**Keywords:** xanthan gum oligosaccharides, seed coating, wheat growth promotion, rhizosphere microbiome, root transcriptomics

## Abstract

Oligosaccharides are plant immune elicitors with recognized roles in seed germination, root development and stress regulation. However, most studies have focused on single oligosaccharides or the independent effects of rhizosphere microorganisms. The coordinated regulation of plant physiological networks and microbial communities by oligosaccharide-based seed coatings remains poorly understood, especially for xanthan oligosaccharides. Here, using wheat root transcriptomics, rhizosphere 16S rRNA sequencing, and physiological assays, we found that a xanthan oligosaccharide composite seed coating significantly improved germination potential, germination rate and seedling vigor. It promoted root and shoot growth under field conditions, optimized the balance of auxin, gibberellin and abscisic acid, activated endosperm hydrolytic enzymes and accelerated nutrient mobilization. Transcriptomics revealed stage-specific regulation of membrane lipid metabolism, hormone signaling, antioxidant defense, carbon/nitrogen metabolism and ABC transporters. Microbiome analysis showed selective enrichment of beneficial genera (*Devosia*, *Paenarthrobacter*, *Citricoccus*), which were positively associated with root nitrogen metabolism and MAPK signaling. Collectively, the coating promoted wheat growth and was associated with stress-related physiological and molecular responses; however, direct stress-challenge and functional-validation experiments are required to verify stress-tolerance mechanisms.

## 1. Introduction

Wheat (*Triticum aestivum* L.) is recognized as one of the most important staple crops worldwide, providing food for over 30 percent of the global population [1]. Seed germination and early seedling establishment are critical developmental stages determining yield potential, and they are regulated by metabolic, cellular, genetic and environmental factors throughout the plant life cycle [2]. These stages are highly susceptible to abiotic stress and environmental fluctuations. Excessive use of chemical seed coatings and fertilizers in modern agriculture has led to soil degradation, microbial imbalance and ecological risks, which severely limit sustainable wheat production. Therefore, the development of green, efficient and environmentally friendly biological seed coatings to enhance germination vigor, seedling growth and stress resistance has become an urgent research priority.

Oligosaccharides such as chitosan oligosaccharides and alginate oligosaccharides have been recognized as effective elicitors of plant immunity. They can activate basal defense responses and are characterized by low toxicity, biodegradability and environmental compatibility. Thus, they have been widely applied in the development of green seed coatings. Previous studies have shown that oligosaccharides can induce reactive oxygen species bursts, defense-related gene expression and secondary metabolite accumulation [3]. They exhibit strong potential in regulating seed germination, root development and stress responses [4]. Seed pretreatment with oligosaccharides has been shown to enhance plant resistance to biotic and abiotic stresses. For example, chitin oligosaccharides can induce plant defense responses, improve stress resilience and promote growth and yield [5,6]. Chitosan oligosaccharides can act as elicitors to enhance plant growth [6,7], disease resistance [8] and salt tolerance [9]. Alginate oligosaccharides can regulate endogenous hormone balance and increase levels of auxin, gibberellin and cytokinin, thereby promoting leaf growth and lateral root development [10]. Despite these advances, most studies have focused on single physiological processes or individual pathways. Systematic understanding of the continuous process from seed germination to seedling establishment and stress response remains limited. In particular, the coordinated regulation among hormone signaling, core metabolic pathways and defense systems under oligosaccharide treatment has not been fully elucidated.

Xanthan gum oligosaccharides (XOG) are low-molecular-weight functional oligosaccharides obtained from xanthan gum by enzymatic or chemical degradation, consisting of 2–20 monosaccharide units linked by glycosidic bonds. Compared with xanthan gum, XOG exhibits higher solubility and membrane permeability, as well as antibacterial, antioxidant and elicitor activities. Previous studies have also shown that the regulatory effects of oligosaccharides on crop growth, yield, quality, and stress resistance vary depending on their types. The results are not always consistent [11,12]. Compared with chitosan and hyaluronic acid oligosaccharides, the functions and molecular mechanisms of xanthan gum oligosaccharides in regulating plant growth, hormone homeostasis, antioxidant systems and stress resistance remain unclear. Xanthan gum has been reported to improve seed–soil adhesion, enhance water retention around seeds and promote early root formation and plant growth. However, the effects of XOG on plant growth regulation and stress resistance have rarely been investigated [13,14,15]. Therefore, how XOG-based seed coatings regulate endogenous hormone homeostasis, endosperm hydrolysis and core metabolic and defense pathways during seed germination and seedling growth remains unclear.

Plant–microbe interactions play essential roles in plant growth promotion, nutrient acquisition and stress resistance [16]. Beneficial rhizosphere microorganisms such as *Bacillus*, *Pseudomonas* and *Devosia* can enhance nutrient availability through phosphate solubilization, nitrogen fixation and organic matter mineralization. They can also improve plant stress resistance by inducing systemic resistance (ISR) and systemic acquired resistance (SAR) [17]. For example, *Paenarthrobacter* can secrete auxins and siderophores, and it can also enhance tolerance to heavy metal stress [18]. *Sphingobacterium* can promote soil organic matter degradation and support plant carbon metabolism [19]. Recent studies have shifted from single strains to the rhizosphere microbiome as a whole [20]. It has been shown that microbial communities can coordinate plant signaling networks, including hormone signaling, MAPK pathways and antioxidant defense, thereby promoting growth and stress resistance [20,21]. Beneficial microbes can release volatile compounds and lipopeptides to activate plant signaling pathways and optimize carbon and nitrogen metabolism. In turn, plants can modulate rhizosphere microbial communities through root exudates such as oligosaccharides, amino acids and phenolic acids, forming a feedback regulatory network [22]. However, most studies have focused either on the physiological effects of oligosaccharides or on the independent roles of rhizosphere microorganisms. Integrated studies on the coordinated regulation of plant physiological networks and rhizosphere microbial communities by oligosaccharide-based seed coatings are still lacking. In addition, traditional bio-seed coatings mainly rely on direct inoculation of beneficial bacteria such as *Bacillus* and *Lysobacter.* The mechanisms by which oligosaccharide-based coatings regulate microbial communities through nutrient-mediated selection, as well as the stage-specific associations between enriched microbes and root transcriptomes, remain unclear. Moreover, rhizosphere microbial functions may vary across different developmental stages of wheat, but these differences have not been well characterized.

In summary, this study aims to elucidate how a xanthan oligosaccharide-based seed coating enhances wheat growth and potential stress resilience through coordinated regulation of hormone signaling, root transcriptional reprogramming and plant–microbe interactions. A composite seed coating was constructed and its effects on seed germination, seedling growth and potential stress resilience were systematically evaluated. The effects on key root signaling pathways and rhizosphere microbial community structure and function were analyzed. This work provides theoretical and technical support for the development of green seed coatings and stress-resistant wheat cultivation.

## 2. Materials and Methods

### 2.1. Chemicals

Xanthan oligosaccharides (XOG) used in this study were prepared from xanthan gum according to our previously established protocol [23]. Their physicochemical properties were characterized in our previous work using XRD, UV scanning, GPC and LC-QTOF mass spectrometry. Briefly, the XOG fraction had an average molecular weight of approximately 1305 Da and a narrow molecular-weight distribution, with a polydispersity index of 1.07. LC-QTOF analysis showed that XOG mainly consisted of xanthan-derived oligosaccharide units, including tetrasaccharides, pentasaccharides, octasaccharides and decasaccharides, with acetyl and pyruvate substitutions. The monosaccharide constituents included glucose, mannose and glucuronic acid. Assay kits for wheat leaves, including activities of peroxidase (POD), superoxide dismutase (SOD), and catalase (CAT), soluble sugars and soluble proteins, were purchased from Jiangsu Enzymatic Immunity Industry Co., Ltd. (Jiangsu, China). TransScript One-Step gDNA Removal and cDNA Synthesis SuperMix and TransStart Tip Green qPCR SuperMix were obtained from Takara Biomedical Technology Co., Ltd. (Dalian, China). All other chemicals and solvents were of analytical grade.

### 2.2. Preparation of Oligosaccharide-Based Seed Coating and Seed Treatment

A characterized XOG stock solution (4 g/L) was diluted 40-fold with deionized water to obtain a 100 mg/L XOG solution. Carboxymethyl cellulose (CMC, 0.15 g) and pectin (0.15 g) were dissolved in 100 g of deionized water and mixed to obtain a 0.3% wall material solution (W/M). Subsequently, 2.5 mL of the 4 g/L XOG stock solution was mixed with 97.5 mL of wall material solution to prepare the coating solution (WX), resulting in a final XOG concentration of 100 mg/L in the WX formulation. Therefore, the same final XOG concentration was used in the XOG and WX treatments.

Seeds were sterilized with 1% sodium hypochlorite for 15 min and rinsed three times with distilled water. Surface moisture was removed using absorbent paper. Seeds were then immersed in deionized water (CK), wall material solution (WM), XOG solution, or coating solution (WX), followed by mixing and air-drying to allow film formation. For coating quantification, 100 seeds were weighed before coating and again after removal of excess surface coating solution but before air-drying. The wet coating formulation retained per seed was calculated as the mass difference divided by the number of seeds. Each WX-coated seed retained 41.1 ± 3.1 mg of wet coating formulation (n = 100 seeds). Coating efficiency was calculated as follows: coating efficiency (%) = (mass of coating formulation retained by seeds/mass of coating formulation applied) × 100, and the coating efficiency was 89.0 ± 4.2%. Because the WX formulation contained 100 mg/L XOG, the final XOG dose delivered to each seed was estimated to be 4.11 ± 0.31 μg seed^−1^, assuming a coating formulation density of approximately 1.0 g/mL.

### 2.3. Wheat Seed Germination Assay

Seeds (50 per dish) were evenly placed in glass dishes lined with three layers of germination paper and supplemented with 10 mL of distilled water. Three replicates were prepared. Dishes were incubated in a growth chamber (GDN-300B-4) under the following conditions: 12 h of light at 30 °C and 12 h of dark at 25 °C, with a light intensity of 8000 lx and a relative humidity of approximately 60%. Moisture was maintained throughout the experiment. Germinated seeds were counted daily until stabilization. Germination was evaluated according to the official seed-testing method specified by the Association of Official Seed Analysts (1990). Germination potential (GE), germination percentage (GP), germination index (GI), mean germination time (MGT), seedling vigor index I (SVI-I), and seedling vigor index II (SVI-II) were calculated using standard formulas.

### 2.4. Determination of Endogenous Hormones and Endosperm Hydrolytic Enzymes

Endogenous hormone levels (IAA, GA, ABA) and enzyme activities (α-amylase, β-amylase, protease, and β-glucanase) were measured at 24, 36, 48, 60 and 72 h during germination. ELISA kits were used to determine hormone contents. Amylase and β-glucanase activities were measured using commercial kits. Protease activity was determined using the Folin–phenol method. Seed samples (1 g) were ground in liquid nitrogen and extracted with phosphate buffer (pH 7.5). The extract was incubated with 1% casein at 37 °C for 10 min. The reaction was terminated with trichloroacetic acid. Absorbance was measured at 680 nm, and enzyme activity was calculated based on a tyrosine standard curve.

### 2.5. Determination of Endosperm Mobilization Index

After 72 h of germination, endosperm mobilization was evaluated by measuring leachate yield, soluble protein, free amino nitrogen (FAN), β-glucan and soluble sugars including glucose, maltose and maltotriose. All measurements were conducted with at least three biological replicates.

#### 2.5.1. Leachate Yield Determination

Leachate content was determined using a pycnometer method. An empty pycnometer was first weighed (m_0_). Uniform seeds (50 grains, 5.000 g) were rinsed, blotted dry, and incubated in 100.0 mL of deionized water at (25 ± 1) °C in the dark. At 12, 24, 48, and 72 h, samples were gently shaken and centrifuged at 4000 r/min for 10 min at 4 °C. The supernatant was collected as leachate. The pycnometer was cleaned, dried, and filled with the leachate without bubbles, equilibrated in a 20.0 ± 0.1 °C water bath for at least 30 min, and weighed (m_1_). Leachate density (ρ) was calculated as (m_1_ − m_0_)/V. The equivalent sugar concentration was obtained from a sucrose standard curve, and total leachate content was expressed per unit fresh weight of seeds.

#### 2.5.2. Soluble Protein Determination

Soluble protein was determined using the Bradford method. Germinated seeds (0.5 g) were homogenized in pre-cooled phosphate buffer (pH 7.8) under ice bath conditions and centrifuged at 4000 r/min for 10 min at 4 °C. The supernatant was mixed with Bradford reagent, and absorbance was measured at 595 nm after 2 min (BioTek Instruments, Inc., Winooski, VT, USA). Protein content was calculated using a standard curve and expressed as mg/g fresh weight.

#### 2.5.3. Free Amino Nitrogen (FAN) Determination

FAN content was determined using the ninhydrin method. Seed extracts were prepared as described above. A 0.5 mL aliquot of supernatant was mixed with acetate buffer (pH 5.0–6.0) and ninhydrin reagent and heated in a boiling water bath for 15 min. After cooling, ethanol was added to terminate the reaction. Absorbance was measured at 570 nm (BioTek Instruments, Inc., Winooski, VT, USA), and FAN content was calculated using a standard curve.

#### 2.5.4. β-Glucan Determination

β-Glucan content was determined using the Congo red method. Seed samples (1 g) were homogenized in acetate buffer (pH 5.0–5.5) and centrifuged at 10,000–15,000 r/min for 15 min at 4 °C. The supernatant was incubated with β-glucan substrate and Congo red solution at 40–50 °C. Absorbance was measured at 492 nm (BioTek Instruments, Inc., Winooski, VT, USA). The content was calculated based on a β-glucan standard curve.

#### 2.5.5. Soluble Sugar Analysis (HPLC)

Soluble sugars were quantified using an Agilent HPLC system (Agilent Technologies, Inc., Santa Clara, CA, USA) equipped with a refractive index detector (RID). Germinated seeds were ground in liquid nitrogen, and 0.50 g of powdered sample was extracted with 5.0 mL of phosphate buffer (50 mM, pH 7.0) under ice bath conditions and centrifuged at 12,000× *g* for 10 min at 4 °C. The supernatant was filtered through a 0.22 μm membrane filter before HPLC analysis. Separation was performed using an Agilent ZORBAX NH_2_ column (4.6 mm × 250 mm, 5 μm; Agilent Technologies, Inc., Santa Clara, CA, USA) with acetonitrile–water (80:20, *v*/*v*, containing 0.1% formic acid) as the mobile phase. The flow rate was 1.0 mL/min, and column temperature was maintained at 35 °C. The injection volume was 10 μL, and the RID temperature was set at 35 °C. Standard solutions of glucose, maltose, and maltotriose were used for calibration. Glucose, maltose, and maltotriose were identified by comparing their retention times with those of authentic standards. Quantification was performed using external standard calibration curves prepared from serial dilutions of glucose, maltose, and maltotriose standards. Samples were diluted when necessary to ensure that peak areas fell within the linear calibration range. Quantification was performed based on peak areas and standard curves, and soluble sugar contents were expressed as g/L.

### 2.6. Seedling Growth Measurement

Seeds were sown in nursery trays (50 seeds per tray, three replicates) and incubated under the same growth conditions as described above. After 12–15 days, 10 representative seedlings per replicate were selected. Root length and shoot length were measured using a ruler. Fresh weights of roots and shoots were also determined.

### 2.7. Determination of Defense System and Physiological Parameters

After 12 days, fresh leaves were collected. The activities of SOD, POD, and CAT were measured using ELISA kits (Jiangsu Enzyme Immunity Industry Co., Ltd., Yancheng, Jiangsu, China). Chlorophyll content was determined by ethanol extraction and absorbance at 665 and 649 nm. Soluble sugars were measured using the anthrone–sulfuric acid method at 630 nm. Soluble proteins were determined by the Bradford method at 595 nm. All measurements were performed in triplicate.

### 2.8. Field Experiment

The field experiment was conducted in an experimental field located at Lexiang Tianyuan Farm, Cha’an Village, Hongqi Subdistrict, Ganjingzi District, Dalian, Liaoning Province, China, during the spring wheat growing season of 2025. The soil at the experimental site was classified as brown soil loam, with an estimated pH of approximately 6.2. The field experiment was arranged in a randomized complete block design with two blocks. Each block contained one plot for each treatment. Each treatment plot consisted of three wheat rows, with an estimated plot size of 3.0 × 0.6 m. The row spacing was approximately 20 cm, and the sowing density was approximately 200 seeds m^−2^. Wheat cultivar Bainong 4199 was used. Treatments included the control (CK) and coating solution (WX). At 12, 50 and 90 days after sowing, 10 representative plants were sampled from the central row of each plot, and each block was treated as an independent biological replicate. The seedling uniformity, root length, plant height, and biomass were evaluated. Statistical analysis was performed to assess treatment effects across growth stages.

### 2.9. Transcriptome Sequencing and Analysis (RNA-Seq)

Total RNA was extracted from wheat root samples using a TRIzol-based protocol following the manufacturer’s instructions. RNA quality and concentration were assessed using a NanoDrop spectrophotometer (ND-2000; Thermo Fisher Scientific, Waltham, MA, USA), and only high-quality samples (RIN ≥ 7.0; OD260/280 = 1.8–2.2) were used for library construction. cDNA libraries were prepared using the Illumina TruSeq RNA Sample Preparation Kit (RS-122-2001 or RS-122-2002; Illumina, Inc., San Diego, CA, USA). Briefly, mRNA was enriched using oligo(dT) beads, fragmented, and reverse-transcribed into first- and second-strand cDNA. After end repair, A-tailing, adapter ligation, and PCR amplification, library quality and fragment size were verified using an Agilent 2100 Bioanalyzer. Paired-end sequencing (150 bp) was performed on an Illumina NovaSeq platform. Raw reads were processed using fastp to remove adapters, low-quality sequences, and poly-N reads. Clean reads were aligned to the wheat reference genome using HISAT2. Gene expression levels were quantified as FPKM using featureCounts. Differentially expressed genes (DEGs) were identified using DESeq2 with thresholds of |log_2_ fold change| ≥ 1 and adjusted *p* < 0.05. Functional enrichment analyses were performed using the GO and KEGG databases. GO enrichment was conducted using GOseq, and KEGG pathway enrichment was analyzed using KOBAS.

### 2.10. DNA Extraction and 16S rRNA Sequencing

Rhizosphere soil DNA was extracted using a MoBio PowerSoil kit (12888-100; Mo Bio Laboratories, Inc., Carlsbad, CA, USA). The V3–V4 regions of 16S rRNA were amplified using primers 341F and 806R. Sequencing was performed on an Illumina NovaSeq platform (Illumina, Inc., San Diego, CA, USA) to generate paired-end reads. Data analysis was conducted using QIIME2 pipelines. Bacterial relative abundance was calculated from sequencing read counts assigned to each taxon.

Microbial α-diversity was evaluated using the Chao1, Shannon and Simpson indices. The Chao1 index was used to estimate community richness, whereas the Shannon and Simpson indices were used to evaluate community diversity and evenness. Microbial β-diversity was calculated based on Bray–Curtis dissimilarity and visualized using principal coordinate analysis (PCoA) and non-metric multidimensional scaling (NMDS). Differences in bacterial community composition between CK and WX treatments were further assessed at the phylum and genus levels based on relative abundance profiles. Differentially abundant taxa were identified by statistical comparison of relative abundance between treatments, and taxa with marked treatment-associated changes were visualized using heatmaps, stacked bar plots, volcano plots and LEfSe analysis.

### 2.11. Statistical Analysis

All data were expressed as mean ± standard deviation. Differences among groups were analyzed by one-way ANOVA followed by Tukey’s post hoc test. A value of *p* < 0.05 was considered statistically significant. Statistical analyses were performed using SPSS 21.

## 3. Results

### 3.1. Xanthan Oligosaccharide-Based Seed Coating Promotes Wheat Seed Germination and Seedling Growth

**Figure 1:**
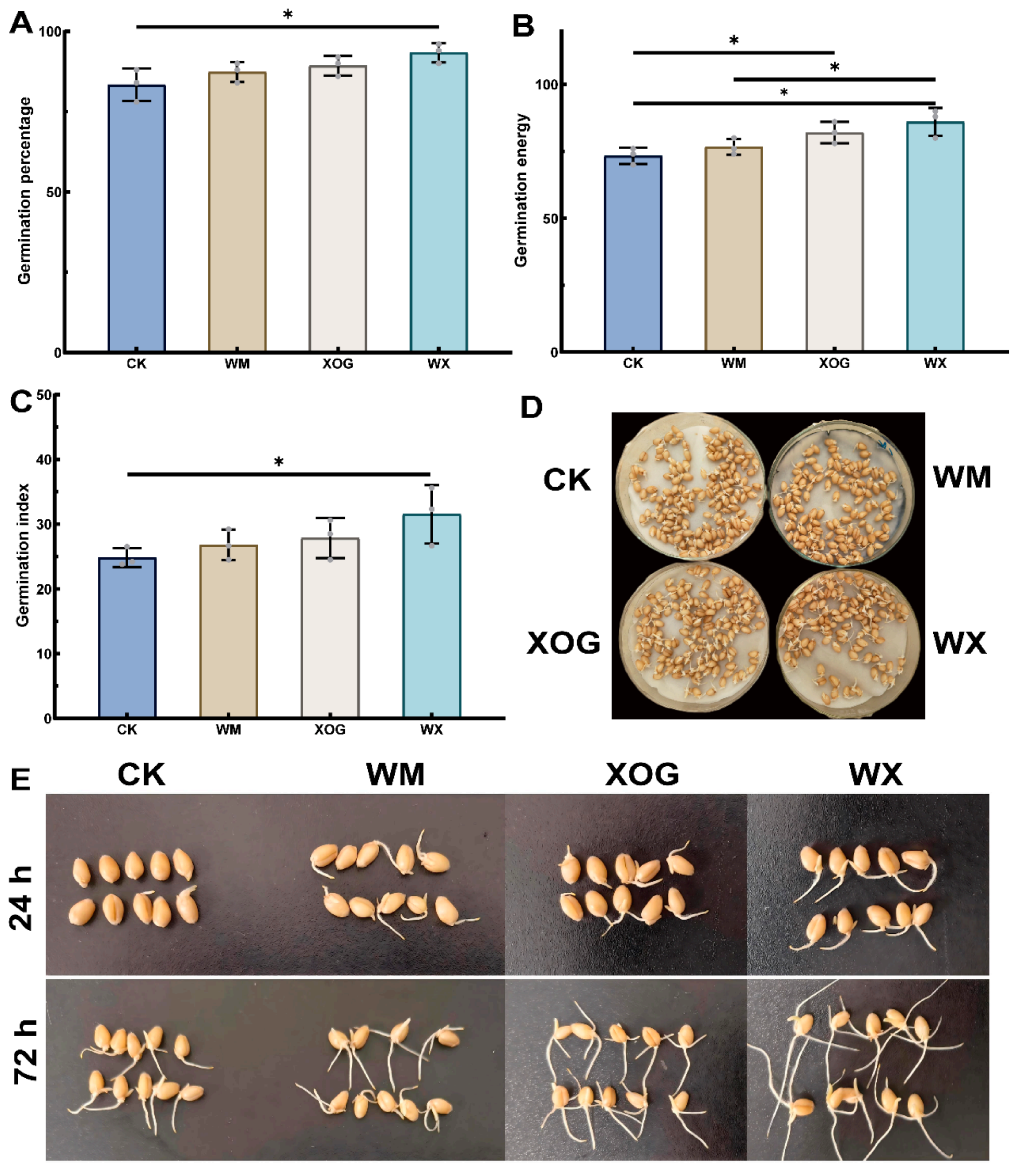
Effects of xanthan oligosaccharide-based seed coating on wheat seed germination and early seedling growth. (**A**) Germination percentage. (**B**) Germination energy. (**C**) Germination index. (**D**) Photographs of seed germination on plates. (**E**) Representative photographs of germinated seeds at 24 h and 72 h post-incubation. Treatments: CK, control (no coating); WM, wall material coating alone; XOG, xanthan oligosaccharide treatment alone; WX, wall material + xanthan oligosaccharide composite seed coating. Data are presented as mean ± standard deviation (n = 3 biological replicates). Asterisks denote significant differences between groups (* *p* < 0.05, one-way ANOVA followed by Tukey’s post hoc test).

The xanthan oligosaccharide-based seed coating significantly enhanced wheat seed germination (Figure 1A–E). Germination percentage represents the final proportion of seeds completing germination. Compared with the control group (CK), the WX group (wall material + XOG) showed a significantly higher germination percentage (Figure 1A), indicating improved seedling establishment and a higher proportion of seeds overcoming dormancy and environmental constraints. Germination energy, which reflects early germination speed and uniformity, was significantly higher in the WX group than in the CK group (Figure 1B). The XOG group also showed higher germination energy than the CK group (Figure 1B), indicating that xanthan gum oligosaccharides are key active substances that accelerate early germination. The germination index (GI), which integrates germination rate and speed, reached the highest value in the WX treatment (Figure 1C), while no significant difference was observed between the XOG and WM groups. This suggests a synergistic effect between xanthan oligosaccharides and the coating matrix in improving overall germination vigor. Germination images (Figure 1D,E) further confirmed that the WX group exhibited the most vigorous root and shoot development and the highest seedling uniformity. Representative photographs at 24 h and 72 h post-incubation (Figure 1E) visually demonstrated that the WX treatment not only accelerated radicle emergence but also promoted more uniform and robust early seedling growth. Overall, xanthan oligosaccharide-based seed coating effectively accelerated germination.

**Figure 2:**
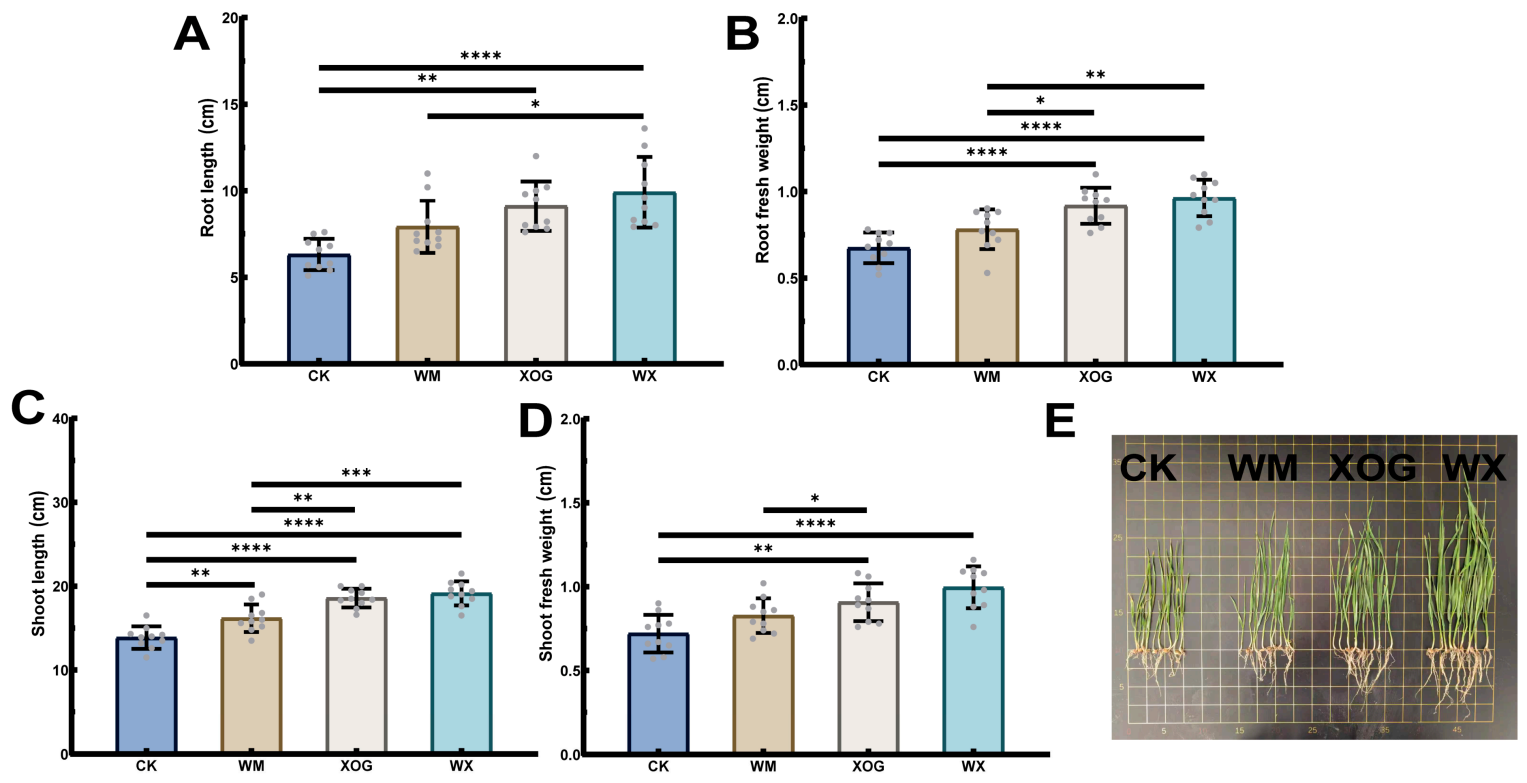
Effects of xanthan oligosaccharide-based seed coating on wheat seedling growth traits under field conditions. (**A**) Root length. (**B**) Root fresh weight. (**C**) Shoot length. (**D**) Shoot fresh weight. (**E**) Representative photographs of whole wheat seedlings at the measured stage. Data are presented as mean ± standard deviation, with individual data points shown (n ≥ 10 biological replicates). Asterisks denote significant differences between groups (* *p* < 0.05, ** *p* < 0.01, *** *p* < 0.001, **** *p* < 0.0001, one-way ANOVA followed by Tukey’s post hoc test).

Seedling growth analysis further showed that XOG-based seed coating significantly promoted early vegetative growth of wheat seedlings, particularly in the WX group (Figure 2A–E). Root development was markedly enhanced. Root length and fresh weight were significantly higher in the WX group compared with the CK and WM groups (Figure 2A,B), while no significant difference was observed between the WX and XOG groups. The root length in the XOG group alone significantly increased compared with the CK and WM groups (Figure 2A), and root fresh weight was also higher than that of the CK group (Figure 2B). These results indicate that xanthan oligosaccharide acts as an effective root growth stimulator, enhancing nutrient acquisition capacity. Although no significant differences in root length and root weight were observed between the WX and XOG groups, both groups had significantly greater root length than the WM group (Figure 2A), highlighting the synergistic role of the coating matrix in optimizing the efficacy of the active oligosaccharides. A similar trend was observed in shoot growth. Shoot length and fresh weight in the WX and XOG groups were significantly increased compared with the CK and WM groups (Figure 2C,D). No significant difference was observed between the WX and XOG groups, indicating that oligosaccharide is the main driver of shoot growth promotion. The coordinated improvement of root and shoot traits indicates a systemic growth response induced by the coating treatment. Representative images (Figure 2E) confirmed more vigorous seedlings in the XOG and WX groups, consistent with quantitative results. Overall, the coating enhanced early seedling vigor, particularly through improved root architecture.

### 3.2. Xanthan Oligosaccharide-Based Seed Coating Improves Field Performance of Wheat

**Figure 3:**
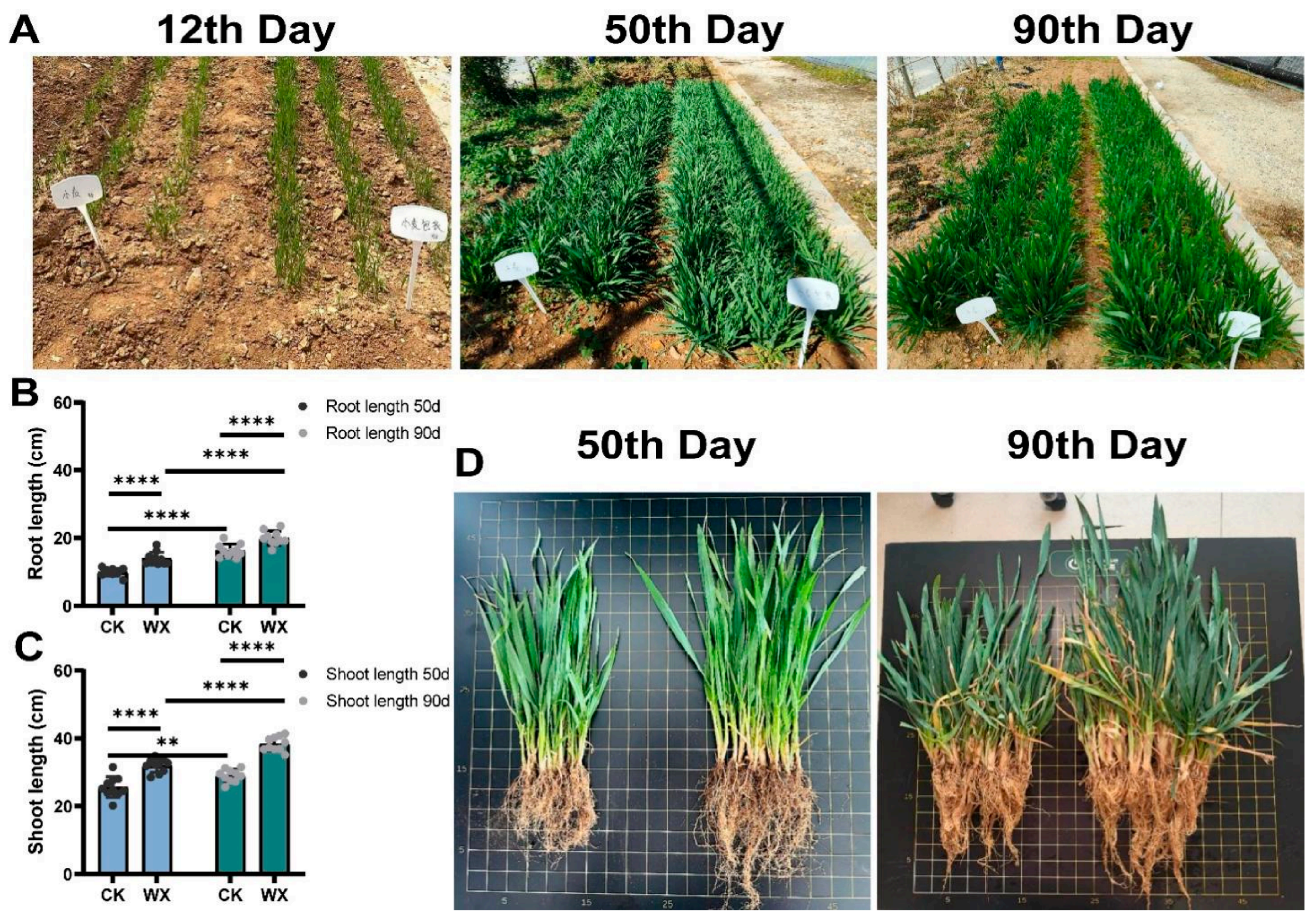
Field performance and growth traits of wheat under CK and WX treatments at different growth stages. (**A**) Representative field photographs of wheat growth at the 12th, 50th, and 90th days after sowing. (**B**) Quantitative analysis of root length in the CK and WX groups at the 50th and 90th days after sowing. (**C**) Quantitative analysis of shoot length in the CK and WX groups at the 50th and 90th days after sowing. (**D**) Representative photographs of whole wheat plants including roots and shoots in the CK and WX groups at the 50th and 90th days after sowing. Data in (**B**,**C**) are presented as mean ± standard deviation (n ≥ 15 biological replicates). Asterisks denote significant differences between groups (** *p* < 0.01, **** *p* < 0.0001, one-way ANOVA followed by Tukey’s post hoc test).

Field experiments confirmed that the xanthan oligosaccharide-based seed coating exerted a persistent growth-promoting effect throughout the wheat growth cycle (Figure 3A–D). The WX treatment consistently produced the most vigorous plants at 12, 50 and 90 days after sowing (Figure 3A). Compared with the CK group, visual evaluation showed improved emergence uniformity, canopy development and biomass accumulation in the WX group (Figure 3A,D). Quantitative measurements at 50 and 90 days further confirmed significant increases in root length and shoot length in the WX group (Figure 3B,C). These results indicate a coordinated improvement of aboveground and belowground traits, reflecting a systemic growth response that supports efficient resource acquisition and yield formation. Overall, the WX treatment conferred sustained agronomic advantages under field conditions.

### 3.3. Regulation of Endogenous Hormone Homeostasis and Endosperm Hydrolysis During Germination

**Figure 4:**
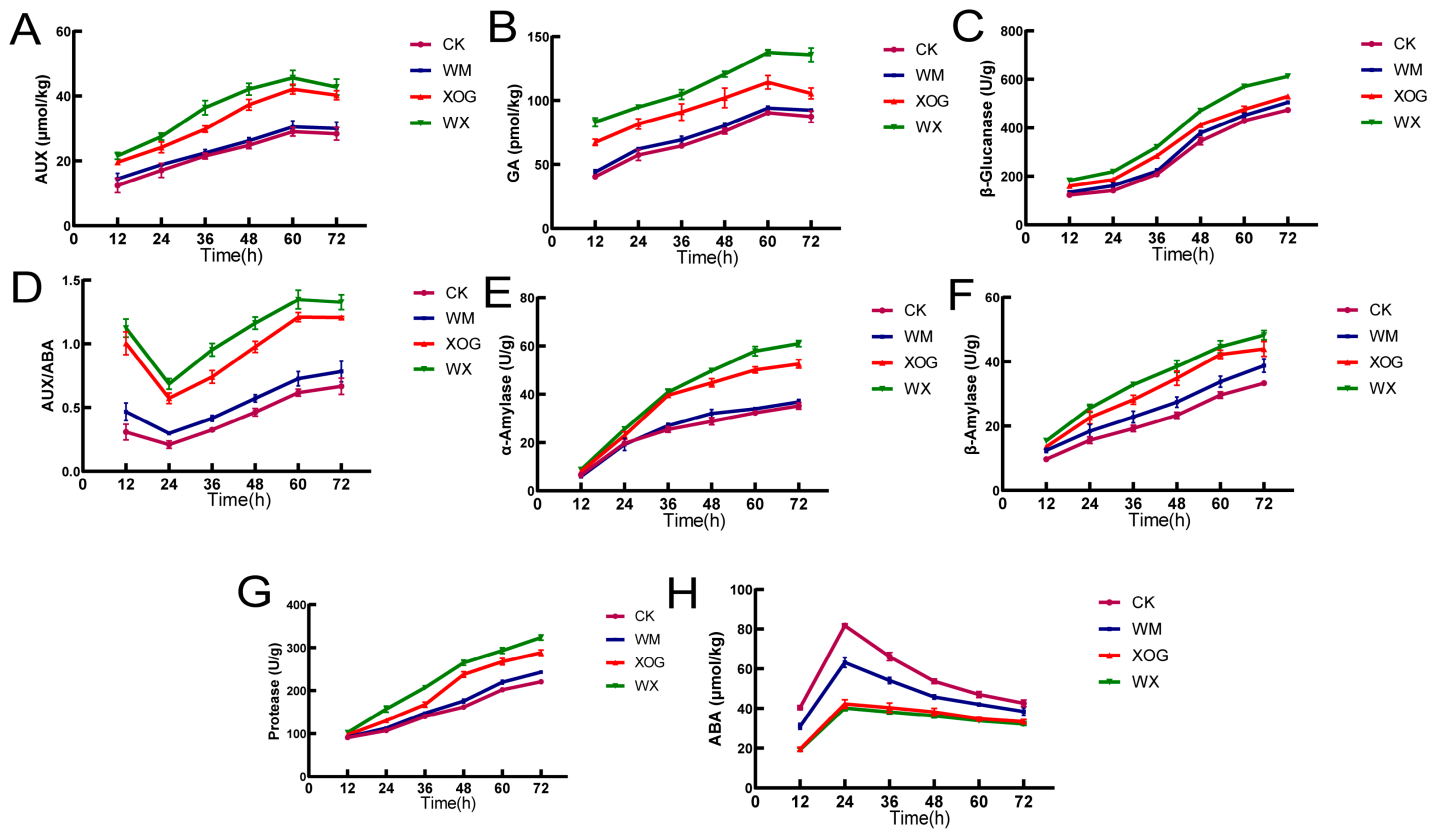
Dynamic changes in endogenous hormone levels and hydrolytic enzyme activities in wheat seeds during germination under different seed coating treatments. (**A**) Auxin (AUX) content. (**B**) Gibberellin (GA) content. (**C**) Abscisic acid (ABA) content. (**D**) AUX/ABA ratio. (**E**) α-amylase activity. (**F**) β-amylase activity. (**G**) Protease activity. (**H**) β-glucanase activity. Data are presented as mean ± standard deviation from three technical replicates. For each treatment and sampling time point, multiple wheat seed samples were pooled and homogenized, and the homogenized sample was divided into three aliquots for independent measurement. Therefore, the error bars represent analytical variation among technical replicates rather than variation among independent biological replicates.

To examine the physiological basis of accelerated germination, endogenous hormones and endosperm hydrolytic enzymes were analyzed (Figure 4A–H). The xanthan oligosaccharide-based coating, particularly the WX coating, was associated with marked changes in the physiological status of wheat seeds. Hormone analysis showed changes in the balance between growth-promoting and growth-inhibiting hormones (Figure 4A–D). Compared with the CK and WM coatings, both WX and XOG coatings increased indole-3-acetic acid (IAA, AUX) and gibberellin (GA) levels during germination (Figure 4A,B), with the most pronounced increase observed in the WX group between 12 and 48 h. In contrast, ABA levels were significantly lower in the WX and XOG groups than in the CK and WM groups throughout germination, although all treatments showed a transient ABA peak at 24 h (Figure 4C). Accordingly, the AUX/ABA ratio decreased briefly at 24 h and then increased progressively. From 36 to 72 h, the XOG and WX groups maintained significantly higher AUX/ABA ratios than the CK and WM groups, with the highest values observed in the WX group during the later germination stage (Figure 4D). This hormonal pattern was consistent with a physiological state favorable for germination and coincided with increased hydrolytic enzyme activities. Concurrently, hydrolytic enzyme activities involved in endosperm mobilization were significantly enhanced (Figure 4E–H). Activities of α-amylase, β-amylase, protease, and β-glucanase were strongly induced in the XOG and WX groups from 24 h onward and increased continuously, reaching a maximum at 72 h (Figure 4E–H). The WX group showed the highest enzyme activities at all time points. These results indicate that the coating synergistically activated starch, protein, and cell wall degradation pathways, thereby accelerating nutrient mobilization. The WX treatment consistently induced the strongest hormonal and enzymatic responses, explaining its superior germination performance. These hormone profiles were consistent with improved germination performance and coincided with increased hydrolytic enzyme activities, but they do not establish a direct causal relationship between hormone changes and enzyme activation.

Compared with the CK and WM groups, endosperm mobilization analysis at 72 h further showed that leachate release was significantly increased in the XOG and WX groups, with no significant difference between the WX and XOG groups (Table 1). Concentrations of soluble proteins and soluble sugars (glucose, maltose and maltotriose) were significantly higher in the XOG and WX groups, with the WX treatment reaching the highest levels. Similarly, the free amino nitrogen content in the leachate of the WX group was significantly higher than that in the other three groups. The XOG group showed a significantly higher free amino nitrogen content than the CK group, but no significant difference was observed between the XOG and WM groups. In contrast, the residual structural component β-glucan content was the lowest in the WX group. Furthermore, the β-glucan content in the XOG group was significantly lower than that in the CK group, while no significant difference was observed between the XOG and WM groups. These results demonstrate that xanthan oligosaccharide-based coating maximized the conversion of endosperm reserves into bioavailable sugars and amino acids, thereby supporting embryo growth.

### 3.4. Xanthan Oligosaccharide-Based Seed Coating Activates the Wheat Defense System and Enhances Seedling Physiological Activity

**Figure 5:**
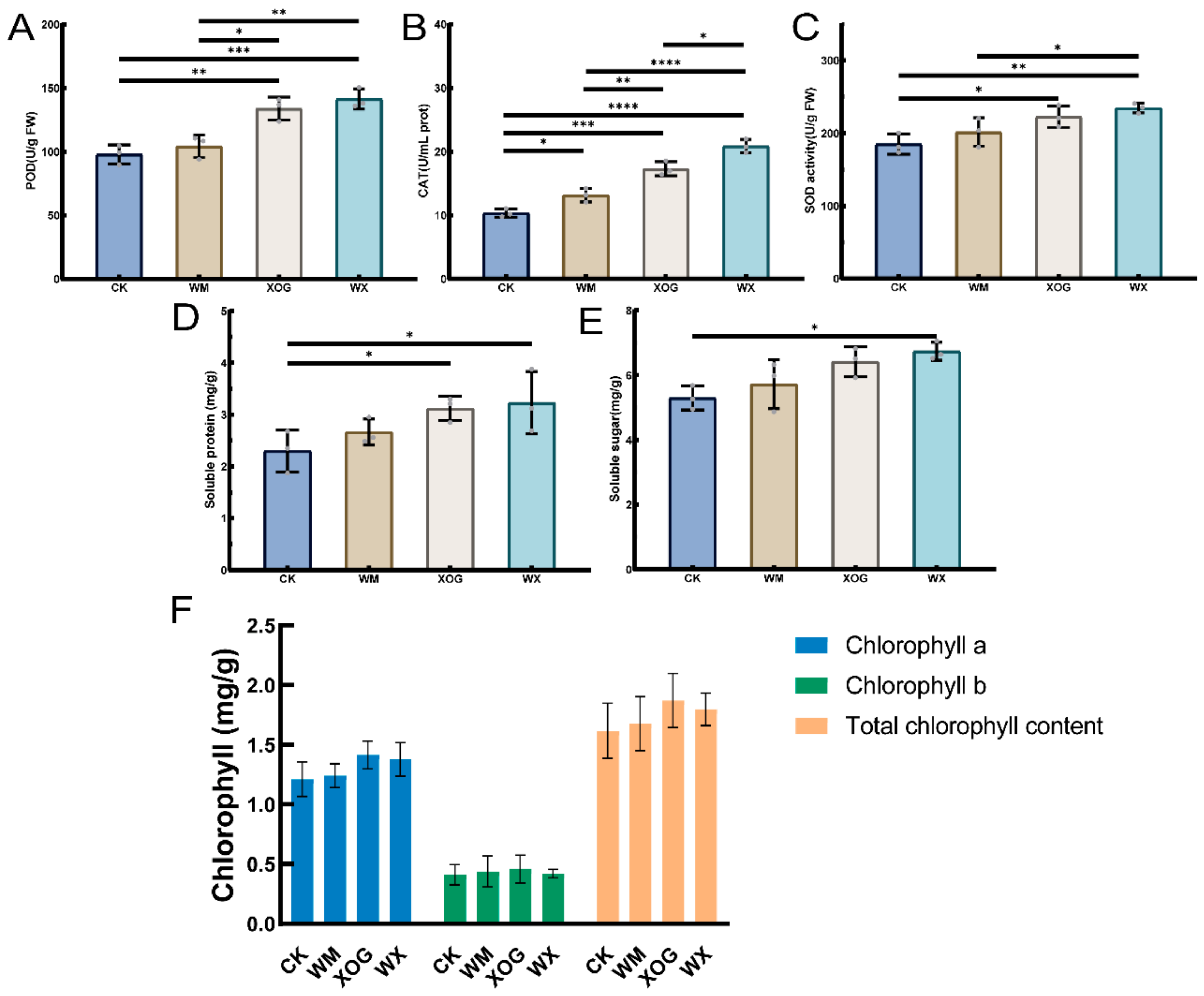
Effects of xanthan oligosaccharide-based seed coating on physiological and biochemical traits of wheat seedlings. (**A**) Peroxidase (POD) activity. (**B**) Catalase (CAT) activity. (**C**) Superoxide dismutase (SOD) activity. (**D**) Soluble protein content. (**E**) Soluble sugar content. (**F**) Chlorophyll a, chlorophyll b, and total chlorophyll content. Data are presented as mean ± standard deviation (n = 3 biological replicates). Asterisks denote significant differences between groups (* *p* < 0.05, ** *p* < 0.01, *** *p* < 0.001, **** *p* < 0.0001, one-way ANOVA followed by Tukey’s post hoc test).

To evaluate the regulatory effects of different treatments on the physiological status of wheat seedlings, antioxidant enzyme activities and key metabolite contents were measured at the late growth stage (Figure 5A–F). Superoxide dismutase (SOD), peroxidase (POD) and catalase (CAT) were identified as key antioxidant enzymes in the stress defense system [24]. POD and CAT activities in seedlings from the XOG group were significantly higher than those in the CK group and the WM group (Figure 5A,B), suggesting that XOG treatment was associated with changes in specific antioxidant enzyme activities. In addition, SOD, POD and CAT activities were all significantly higher in the WX group than in the CK and WM groups (Figure 5A–C), while CAT activity in the WX group was significantly higher than that in the XOG group (Figure 5B). These results indicate that the composite coating was associated with stronger antioxidant enzyme responses than the single XOG treatment for some measured traits. However, these enzyme activity data alone do not demonstrate the establishment of a complete defense system or a causal stress-resistance mechanism.

Soluble sugars and soluble proteins, as major osmotic regulators and metabolic reserves, not only maintain cellular turgor pressure and water balance under stress conditions but also serve as readily available carbon and nitrogen sources, providing substrates for metabolism and biosynthesis [25]. Consequently, they contribute directly to enhanced seedling vigor and potential stress resilience. In the WX group, the accumulation of soluble sugars and soluble proteins was significantly higher than that in the CK group (Figure 5D,E), while no significant differences were observed compared with the XOG and WM groups. In contrast, the XOG treatment induced a significant increase only in soluble protein content (Figure 5D). These results indicate that xanthan oligosaccharides effectively promote the synthesis of osmotic regulatory substances and metabolic reserves, whereas the composite seed coating further amplifies this effect, thereby providing a stronger physiological basis for maintaining turgor pressure, water balance, and rapid growth under stress conditions.

Notably, no significant differences were observed in chlorophyll a, chlorophyll b, or total chlorophyll content among all treatments (Figure 5F). This confirms that xanthan oligosaccharide-based seed coatings enhance seedling vigor primarily through modulation of defense systems and metabolic regulation, rather than by altering baseline photosynthetic capacity during development. In summary, xanthan oligosaccharides provide a key “defense signal,” whereas the composite seed coating induces physiological changes associated with antioxidant capacity and osmotic/metabolic adjustment, which may contribute to improved seedling vigor under the tested growth conditions.

### 3.5. Global Transcriptomic Profiling and GO Enrichment Analysis of Wheat Root Transcriptome

**Figure 6:**
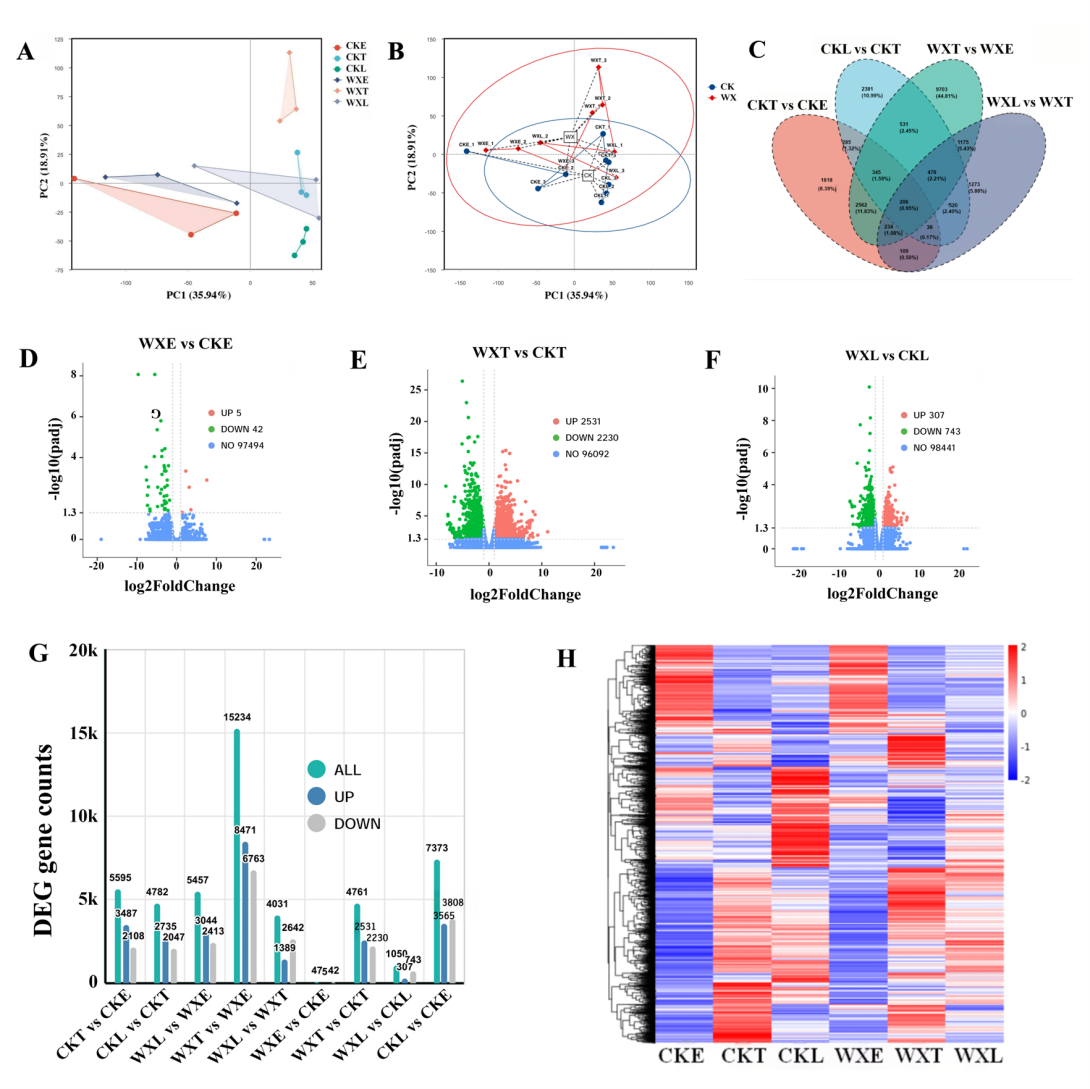
Overview of transcriptomic profiles in wheat seedlings under CK and WX treatments across developmental stages. (**A**,**B**) Principal coordinate analysis (PCoA) showing transcriptional separation between the CK and WX groups across stages. (**C**) Venn diagram illustrating shared and unique differentially expressed genes (DEGs) across treatments and stages. (**D**–**F**) Volcano plots of DEGs in pairwise comparisons (CK vs. WX) at different stages, with significantly upregulated (green), downregulated (red), and non-significant (blue) genes. (**G**) Bar chart showing the number of upregulated, downregulated, and total DEGs in all pairwise comparisons. (**H**) Heatmap showing the expression profiles of core DEGs across all samples. Stages: E, emergence stage; T, two-leaf stage; L, tillering stage.

Global transcriptomic profiling was performed to characterize transcriptional variation in wheat roots between the CK and WX treatments at the emergence stage (E), two-leaf-one-heart stage (T), and late growth stage (L) (Figure 6A–H). PCoA analysis showed that the samples were primarily separated according to developmental stage, while partial treatment-associated separation between CK and WX samples was observed within specific stages (Figure 6A,B). The clustering of biological replicates from the same treatment and developmental stage supported the acceptable reproducibility of the transcriptomic dataset. Dynamic analysis of differentially expressed genes (DEGs) across developmental stages revealed clear stage specificity and continuity in the transcriptional responses of wheat roots. The most extensive transcriptional reprogramming occurred during the transition from the emergence stage to the two-leaf-one-heart stage. Specifically, 10,579 DEGs were identified in the WXE vs. WXT comparison, which was approximately 2.7-fold higher than the number of DEGs detected in the CKE vs. CKT comparison. Volcano plot analysis further showed that, during the transition from E to T, the proportion of upregulated genes was higher than that of downregulated genes in both the WX and CK groups, with a more pronounced upward bias in the WX group. Within the WX treatment, differential gene expression was most active at the two-leaf-one-heart stage, where 4757 DEGs were detected. This number was approximately fourfold higher than that observed at the late growth stage, whereas only a limited number of DEGs were detected at the emergence stage. These results indicate that the transcriptional response associated with the xanthan oligosaccharide-based composite seed coating was most evident at the two-leaf-one-heart stage. In addition, a core response set consisting of 206 genes was identified, and these genes were continuously regulated by the WX treatment across all developmental stages. The WXE vs. WXT comparison also showed the highest proportion of stage-specific DEGs, accounting for approximately 44.81%, further supporting the two-leaf-one-heart stage as a critical window for transcriptional reprogramming. Overall, these results indicate that the xanthan oligosaccharide-based seed coating was mainly associated with broad transcriptional regulation at the two-leaf-one-heart stage and with the sustained regulation of a core set of responsive genes, which may contribute to the developmental and adaptive responses of wheat seedlings.

**Figure 7:**
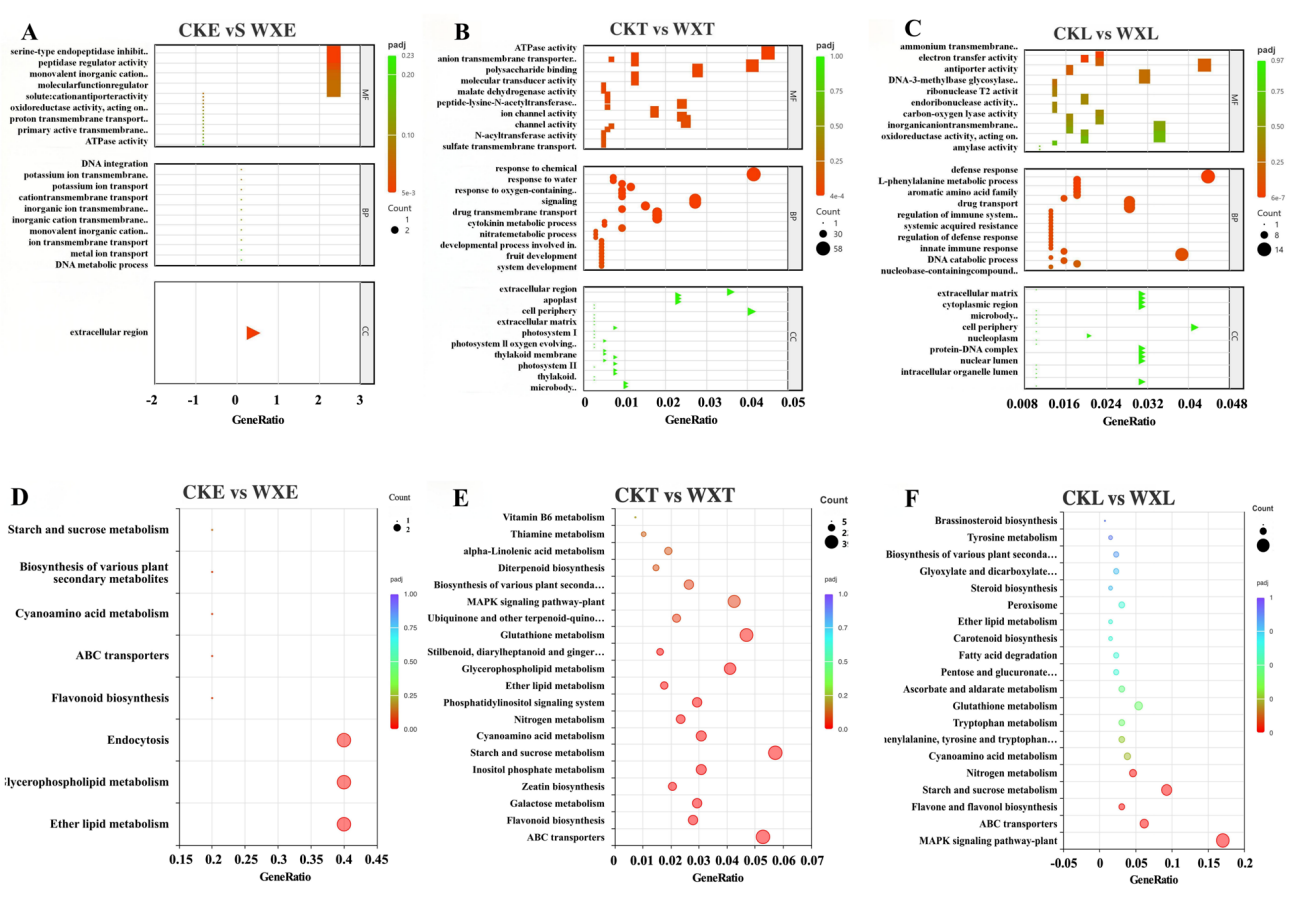
GO and KEGG enrichment analyses of differentially expressed genes in wheat roots under CK and WX treatments across developmental stages. (**A**–**C**) GO enrichment analysis of DEGs at the emergence, two-leaf, and late growth stages, respectively. (**D**–**F**) KEGG enrichment analysis of DEGs at the emergence, two-leaf, and late growth stages, respectively. In the GO and KEGG enrichment bubble plots, dot size represents the number of DEGs enriched in each term or pathway, and dot color represents the adjusted *p* value. Warmer colors indicate lower adjusted *p* values and stronger enrichment significance. Stages: E, emergence stage; T, two-leaf stage; L, late growth stage.

GO enrichment identified stage-specific groups of DEGs annotated to extracellular protease inhibition, ion transport, hormone metabolism, signal transduction, and defense-related processes (Figure 7A–C). At the emergence stage, WX-induced differentially expressed genes (DEGs) were mainly associated with extracellular-region homeostasis-related GO terms, including extracellular region, serine-type endopeptidase inhibitor activity, peptidase regulator activity, and monovalent inorganic cation transmembrane transporter activity (Figure 7A). Most of these genes were significantly downregulated, indicating that the WX treatment modulated early root–soil interface homeostasis and suppressed unnecessary basal stress-related activities during initial establishment. At the two-leaf stage, the WX treatment resulted in a marked expansion of enriched functional categories, including environmental-sensing terms, such as response to chemical, response to water, response to acid chemical, response to oxygen-containing compound, and response to abiotic stimulus; hormone-related terms, including regulation of hormone levels and hormone metabolic processes; and signal transduction and transport terms, including inorganic anion transmembrane transporter activity, active transmembrane transporter activity, and phosphorelay signal transduction system (Figure 7B). Compared with the CK group, genes associated with core signal transduction systems and active transmembrane transport functions were generally upregulated in the WX group. In contrast, most genes involved in direct stress-response pathways were significantly downregulated. This pattern suggests that the WX treatment shifted root physiology from passive environmental sensing toward enhanced internal regulatory optimization and efficient signal integration. At later developmental stages, WX-induced enriched pathways were mainly related to defense response, L-phenylalanine biosynthetic process, aromatic amino acid family biosynthetic process, aromatic amino acid family metabolic process through the prephenate pathway, ammonium transmembrane transporter activity, and prephenate dehydratase activity (Figure 7C). Although most genes within these pathways remained downregulated, their enrichment indicated a strategic allocation of metabolic resources toward defense-related readiness rather than sustained high-level activation. Overall, GO enrichment analysis suggested that the WX treatment was associated with a developmental transition in wheat roots, shifting from early establishment of basal physiological homeostasis to enhanced adaptive regulation at the two-leaf stage, and finally to a balanced state characterized by defense preparedness and metabolic stabilization.

### 3.6. KEGG Enrichment Analysis of Wheat Root Transcriptome

KEGG enrichment analysis suggested staged transcriptional changes associated with the WX treatment (Figure 7D–F). At the emergence stage, DEGs were mainly enriched in membrane lipid metabolism and transport pathways. Most membrane lipid metabolism-related DEGs were downregulated, whereas ABC transporter-related DEGs were mainly upregulated (Figure 7D), indicating a preferential enhancement of transmembrane transport establishment rather than global metabolic enrichment. At the two-leaf stage, enriched pathways were significantly expanded, covering primary metabolism, signal transduction, and defense-related processes (Figure 7E). Nitrogen metabolism and flavonoid biosynthesis genes were notably upregulated, indicating coordinated enrichment of nutrient acquisition, growth, and stress preparedness. At the late stage, enriched pathways became more concentrated, with MAPK signaling emerging as the core regulatory module, while carbon–nitrogen metabolism and flavonoid biosynthesis remained enriched but were overall downregulated (Figure 7F), suggesting a shift toward signal-centered defense integration and metabolic stabilization. Overall, the WX treatment induced staged regulation characterized by early transport enrichment, mid-stage metabolic signaling enrichment, and late-stage defense coordination (Figure 7D–F).

**Figure 8:**
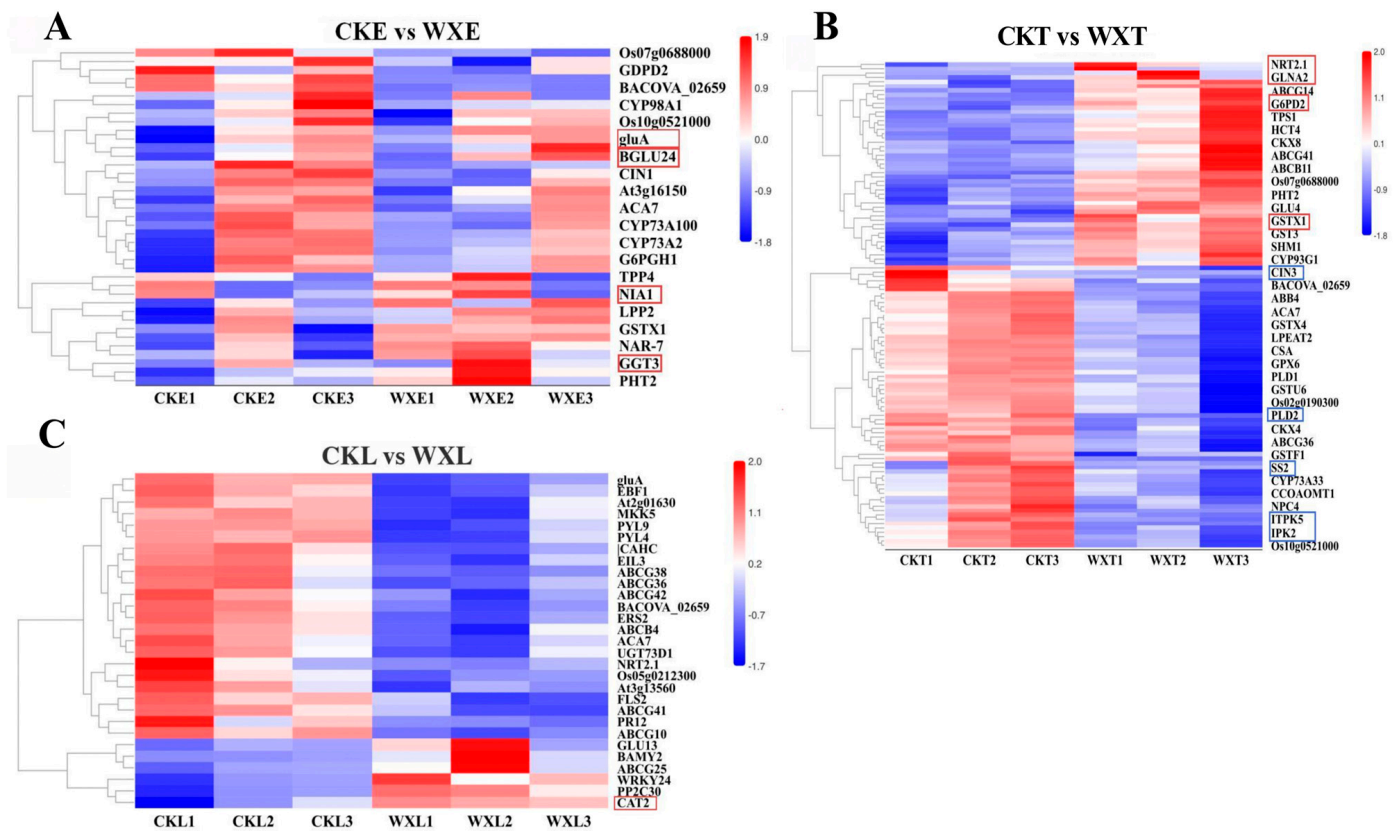
Expression patterns of core genes associated with key functional pathways in wheat roots under CK and WX treatments. (**A**–**C**) Heatmaps showing the expression patterns of core genes involved in key functional pathways at the emergence, two-leaf, and late growth stages, respectively. In the heatmaps, red and blue indicate relatively high and low row-scaled normalized expression levels, respectively, for each gene across samples. CKE, control group at the emergence stage; CKT, control group at the two-leaf stage; CKL, control group at the late growth stage; WXE, WX-treated group at the emergence stage; WXT, WX-treated group at the two-leaf stage; WXL, WX-treated group at the late growth stage. The numbers 1, 2, and 3 following each sample abbreviation indicate three independent biological replicates within the corresponding treatment and developmental stage.

Based on these stage-specific transcriptional patterns, four major functional modules were further analyzed, including membrane lipid metabolism and membrane transport pathways, antioxidant defense-related pathways, hormone-related signaling pathways and ABC transporter pathways. The expression patterns of core genes in these pathways across different developmental stages are visualized in heatmaps (Figure 8A–C).

#### 3.6.1. Phospholipid Metabolism Pathway

In the phospholipid metabolic pathway, multiple homologs of phospholipase Dα2 (*PLD2*) and another lipid hydrolase gene (*Os05g0495700*) were consistently downregulated in the WX group at both the emergence and two-leaf stages (Figure 8A,B). PLDα2, a key enzyme in stress responses, generates phosphatidic acid (PA) as a stress signal [26]. The sustained suppression of these genes suggests that the WX treatment may attenuate core stress signaling or reduce unnecessary PA accumulation in early roots, thereby preserving membrane integrity and reallocating resources toward growth. These expression patterns may be consistent with altered lipid-signaling status; however, direct measurements of membrane lipid composition, phosphatidic acid levels and membrane integrity are required to test this hypothesis. Conversely, two negative regulatory genes (*LPP2* and *Os07g0688000*) were consistently upregulated. LPP2 encodes a lipid phosphate phosphatase that degrades PA, terminating its signal [27]; the homolog of *Os07g0688000* is a leucine-rich repeat receptor-like kinase involved in developmental signal perception [28]. Their upregulation, together with *PLD2* downregulation, may synergistically dampen stress signals and promote programmed root development. At the two-leaf stage, the WX treatment specifically induced additional genes involved in membrane synthesis and remodeling, including nonspecific phospholipase C (*NPC2*), cardiolipin synthase (*taz*), *NMT2* and *PEAM2*. Their upregulation points to active biogenesis and remodeling of specialized membrane systems to support heightened demands for transport, energy conversion, and signaling during rapid seedling growth.

#### 3.6.2. Key Antioxidant Defense Pathways

KEGG enrichment analysis indicated that the oligosaccharide-based seed coating agent (WX) stage-dependently regulated core antioxidant defense pathways, including phenylpropanoid metabolism, glutathione metabolism, and the MAPK signaling pathway in plants. These coordinated regulations collectively supported redox homeostasis and potential stress resilience during root development. At the emergence stage, the WX treatment rapidly established a glutathione-based reactive oxygen species (ROS) scavenging system. In the glutathione metabolism pathway, genes responsible for NADPH supply (*G6PD2*) and glutathione biosynthesis (*GSTX1* and *GGT3*) were significantly upregulated. In contrast, the energy-intensive upstream branch of the phenylpropanoid pathway was suppressed, as key rate-limiting enzyme genes (e.g., *CYP73A* family) and lignin biosynthesis-related gene *CCOAOMT1* were significantly downregulated. This suggests that secondary metabolism was not fully activated at this stage. In the MAPK and hormone-related signaling pathways, several negative regulators of ABA and ethylene signaling (e.g., *PYL9*, *PYL4*, and *ERS2*) were downregulated, whereas ABA-related protein phosphatase 2C genes (*PP2C30* and *PP2C50*) and the stress-responsive transcription factor WRKY24 were upregulated. These results suggest that basal stress perception was attenuated, allowing greater allocation of resources to early establishment and primary defense.

At the two-leaf-one-heart stage, antioxidant defense capacity was systematically enhanced in WX-treated roots. Phenylpropanoid metabolic flux was redirected toward the synthesis of high-value defensive metabolites, while the glutathione system was continuously strengthened and functionally reprogrammed. In the phenylpropanoid pathway, upstream core biosynthetic genes (*CYP73A* family) remained suppressed, whereas downstream branches were selectively activated. Genes encoding key enzymes for proanthocyanidin biosynthesis (ANR), phenolic amide formation (*HCT4*), lignin monomer hydroxylation (*CYP98A1*), and flavonoid modification (*CYP75A5* and *CYP93G1*) were significantly upregulated. This regulatory pattern indicates enhanced accumulation of antioxidant flavonoids and proanthocyanidins, contributing to the formation of a soluble chemical antioxidant barrier in seedlings. In glutathione metabolism, NADPH-regenerating *G6PD2* genes were significantly upregulated, indicating a strengthened reducing power supply. However, a pronounced functional shift was observed, as more than ten glutathione S-transferase (*GST*) genes (e.g., *GSTU6* and *GSTF1*) were broadly downregulated. Given that GSTs consume glutathione for detoxification conjugation, their downregulation suggests a strategic reduction in broad-spectrum detoxification capacity during a critical developmental stage. Instead, glutathione may have been preferentially allocated to direct ROS scavenging or to biosynthetic processes such as phenolic compound production. This reflects an optimized allocation and prioritization of defense functions under resource-limited conditions.

At the late growth stage, the regulatory focus of the antioxidant system shifted toward signaling integration and homeostatic maintenance (Figure 8C). The MAPK signaling pathway became a central regulatory hub in the WX-treated roots. While ABA and ethylene receptor signaling remained suppressed, *CAT2* expression was further significantly upregulated, enhancing direct H_2_O_2_ detoxification capacity. In contrast, genes associated with pattern-triggered immunity, such as *FLS2* and *PR12*, and the MAPK kinase MKK5 were downregulated, suggesting a reduced basal immune alert state after establishment of a new physiological equilibrium. Consistently, genes involved in downstream phenylpropanoid modification and transport, including *UGT73D1* and *ABCG42*, were also downregulated, indicating that active synthesis and secretion of defense metabolites had stabilized and shifted toward storage or utilization. Overall, the WX treatment coordinated a multilayered defense network in wheat roots, integrating metabolic synthesis, redox balance, and signal transduction, thereby providing a comprehensive molecular explanation for the enhanced potential stress resilience observed in seedlings.

#### 3.6.3. Plant Hormone Signaling and Intracellular Second Messenger Pathways

KEGG pathway enrichment analysis further revealed the central regulatory roles of plant hormone signaling and intracellular second messenger pathways in wheat root development. Cytokinin was identified as a key hormone regulating cell division and differentiation, and it acts synergistically with gibberellin and auxin to promote germination [29]. At the emergence stage, genes encoding cytokinin degradation enzymes (*CKX3* and *CKX8*) and a key enzyme for the biosynthesis of the active cytokinin form (tZ-type), CYP735A2, were significantly upregulated in the WX group. In contrast, multiple glycosyltransferase genes involved in cytokinin inactivation (*CISZOG1*) were significantly downregulated. This regulatory pattern was maintained and further extended at the two-leaf-one-heart stage. In addition to the sustained upregulation of *CKX3*, *CKX8*, and *CYP735A2*, *IPT5*, which encodes a rate-limiting enzyme in cytokinin biosynthesis, and another degradation-related gene (*CKX11*) were significantly upregulated under the WX treatment. Meanwhile, additional *CISZOG1* genes and another degradation-associated gene (*CKX4*) were significantly downregulated. These results indicate that WX treatment altered the transcriptional state of genes associated with cytokinin biosynthesis, which may be related to cell division and differentiation in the root apical meristem and may provide a potential hormonal basis for the observed root growth-promoting phenotype.

The two signaling pathways were generally suppressed at the emergence stage but were selectively activated at the two-leaf-one-heart stage. At emergence, multiple kinase genes involved in signal synthesis (e.g., *ITPK4/5*, *IPK2*, and *PIP5K9*) were downregulated in the WX group compared with the control, indicating suppression of basal signaling activity. At the two-leaf-one-heart stage, genes responsible for generating the key phosphatidylinositol-4,5-bisphosphate (*PIP2*) signaling platform *(PIP5K9* and *PIP5K2*) were specifically upregulated. In contrast, most genes involved in downstream second messenger production pathways, including IP3/DAG generation via *PLC6*, PA production via *DGK1*, and PIP3 synthesis via *FAB1D*, were broadly downregulated. This pattern suggests a controlled signaling strategy that may prevent excessive signal amplification or metabolic dissipation. In addition, the upregulation of the calmodulin-like protein gene *CML9* may contribute to the decoding of limited calcium signaling events. Overall, the WX treatment coordinately enhanced cytokinin biosynthesis and finely reshaped the phosphatidylinositol signaling network, thereby establishing an efficient hormone-driven and signal transduction system that supports early root development in wheat.

#### 3.6.4. ABC Transporters Pathways

ABC transporters are responsible for the transmembrane transport of diverse substrates, including phytohormones and secondary metabolites, and play essential roles in plant growth, development and stress adaptation. At the emergence stage, key hormone transporters were regulated by the WX treatment. Compared with the control, ABCG25, which encodes an abscisic acid (ABA) efflux transporter, and multiple ABCB11 homologs involved in auxin export were significantly upregulated in the WX group. In contrast, genes associated with lipid transport (*ABCG48*) and hormone transport regulation (e.g., *ABCG38* and *ABCB4*) were significantly downregulated. These results indicate that WX treatment increased the transcript abundance of genes annotated as ABA- and auxin-related ABC transporters, suggesting candidate changes in hormone-transport capacity that require direct transport validation.

At the two-leaf-one-heart stage, as the demand for transmembrane transport increased markedly, transcriptional regulation of the ABC transporter family exhibited strong functional divergence. On one hand, genes involved in cytokinin uptake (*ABCG14*), auxin export (*ABCB11*), and ABA export (*ABCG25*) were continuously and significantly upregulated, suggesting enhanced coordination of multiple hormonal signals to support rapid growth. On the other hand, more than 20 genes were significantly downregulated in the WX group, including several PDR-type ABCG transporters (e.g., *ABCG36*, *ABCG37*, and *ABCG39*) that are potentially involved in secondary metabolite efflux, as well as lipid metabolism-related genes such as *ABCD1*. This broad suppression of transport activity was consistent with the concurrent enhancement of phenylpropanoid-derived defense metabolism, suggesting a coordinated intracellular retention of defensive metabolites. As a result, an efficient intracellular chemical defense system was likely established rather than promoting metabolite export.

At the late growth stage, transcriptional changes in ABC transporter genes were markedly reduced, and regulatory focus became more centralized. Notably, *ABCG25* expression remained continuously upregulated across all three stages, suggesting a sustained role in ABA transport and systemic stress adaptation throughout development. In contrast, multiple ABCG family members (e.g., *ABCG36*, *ABCG38*, and *ABCG41*) and *ABCB4* were downregulated, reflecting a return of transmembrane transport activity to basal homeostasis after the active growth and biosynthetic phases. Overall, the WX treatment reprogrammed the temporal expression patterns of ABC transporter genes across developmental stages, thereby dynamically regulating hormone distribution and defense metabolite allocation in wheat roots.

#### 3.6.5. Carbon and Nitrogen Metabolic Pathways

KEGG analysis indicated that the WX treatment significantly regulated core carbon and nitrogen metabolic pathways during root development, including starch and sucrose metabolism, cyanogenic amino acid metabolism and nitrogen metabolism. At the emergence stage, both carbon mobilization and nitrogen assimilation were enhanced by the WX treatment. In the starch and sucrose metabolism pathway, genes encoding β-glucosidases (*BGLU16/24/25*) and glucose-releasing enzyme (*gluA*) were upregulated in the WX group, whereas sucrose synthase (*SUS4*) and starch synthase (*SS2*) were downregulated. These results indicate that carbon flux was redirected toward the degradation of storage carbohydrates to support energy supply. In the concurrently activated nitrogen metabolism pathway, nitrate reductase (*NIA1*), nitrate transporter (NRT2.1) and glutamine synthetase (*GLNA2*) were upregulated, indicating enhanced nitrogen uptake and assimilation. In addition, genes involved in cyanogenic amino acid metabolism, including β-glucosidase and γ-glutamyl transpeptidase (*GGT3*), were also upregulated, suggesting potential roles in detoxification and nitrogen/sulfur turnover. Collectively, these changes provided initial metabolic support for the transition from heterotrophic to autotrophic growth.

At the two-leaf-one-heart stage, carbon and nitrogen metabolism underwent large-scale reprogramming characterized by coordinated activation and restriction. Starch and sucrose degradation were further enhanced, as additional genes encoding BGLUs, β-amylase (*BAM3*), and trehalose-6-phosphate synthase (*TPS1*) were upregulated, while biosynthetic (*SS2*) and sucrose cleavage pathways (*CIN1/2/3* and *SUS4*) were broadly suppressed. This pattern likely ensured stable sugar supply and transport. Meanwhile, core nitrogen assimilation genes (*NIA1*, *NRT2.1*, and *GLNA2*) remained highly active. The activation scope of cyanogenic amino acid metabolism was further expanded under the WX treatment, involving glycosylation-related (*UGT85B1*) genes and one-carbon metabolism-related (*SHM1*) genes, suggesting deeper integration into nitrogen metabolism and secondary biosynthetic networks, thereby providing metabolic precursors for rapid growth.

At the late growth stage, metabolic activity was attenuated and shifted toward homeostatic maintenance. Starch and sucrose metabolism were generally reduced, and most carbohydrate-degrading genes (e.g., *gluA*) were downregulated, indicating decreased reliance on stored carbon sources. In addition, key nitrogen assimilation genes (e.g., *NRT2.1*) and a calcium transporter gene (*ACA7*) were downregulated, suggesting a transition from rapid assimilation to storage or recycling of nitrogen resources. Overall, the WX treatment dynamically regulated carbon and nitrogen metabolic networks, enabling rapid activation at the emergence stage, high metabolic support during vigorous growth, and a transition toward homeostasis at the late stage. This coordinated regulation ensured the stage-specific energy and structural demands of root development and contributed to enhanced seedling performance.

### 3.7. Xanthan Oligosaccharide-Based Seed Coating Reshaped the Structure and Function of Wheat Rhizosphere Microbial Communities

**Figure 9:**
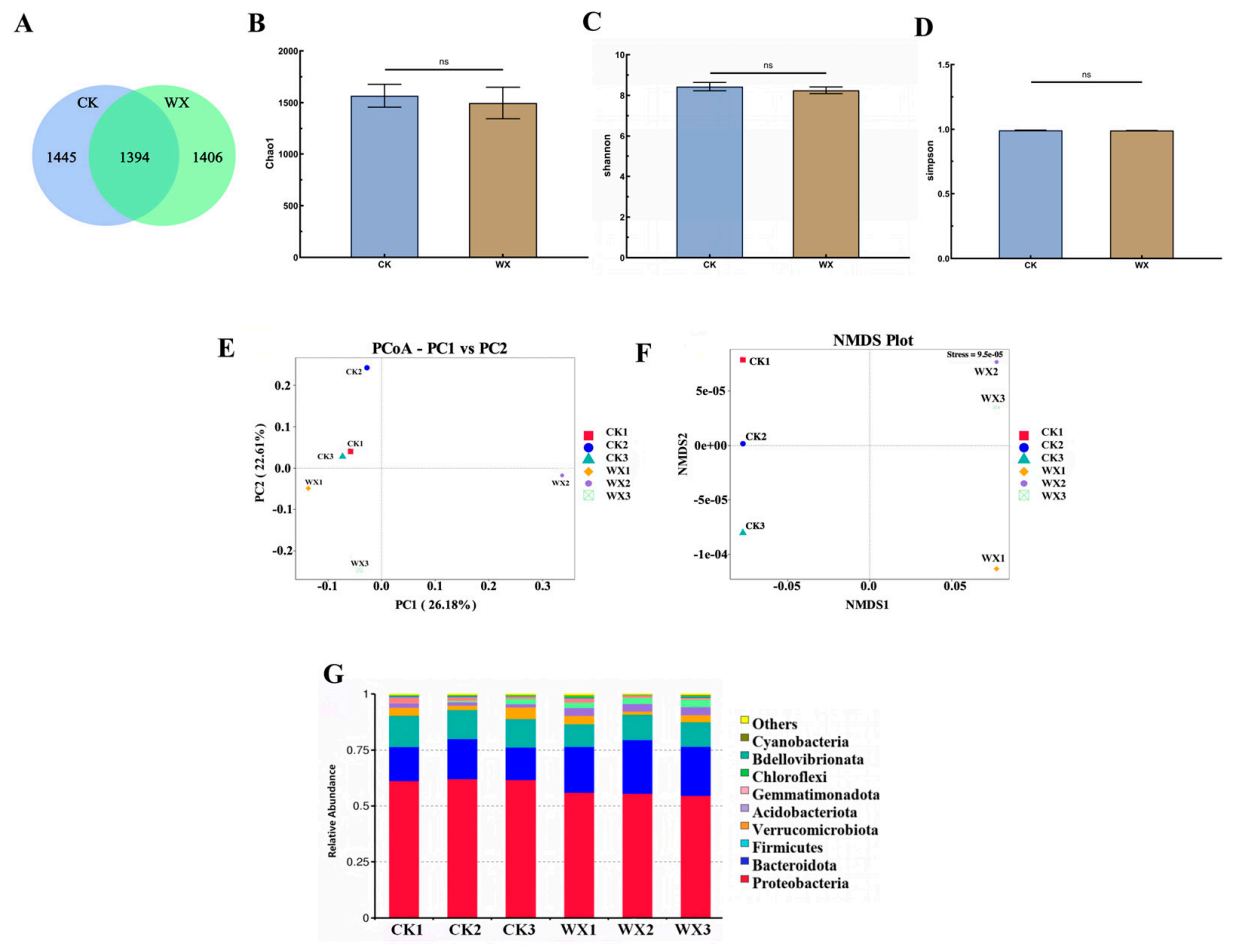
Effects of xanthan oligosaccharide-based seed coating on wheat rhizosphere bacterial community structure. (**A**) Venn diagram showing the shared and unique operational taxonomic units (OTUs) between the CK and WX groups. (**B**–**D**) Alpha diversity indices: Chao1 ((**B**), richness), Shannon ((**C**), diversity), and Simpson ((**D**), evenness) indices (ns, no significant difference, Student’s *t*-test). (**E**) Principal coordinate analysis (PCoA) based on Bray–Curtis distance showing β-diversity of bacterial communities. (**F**) Non-metric multidimensional scaling (NMDS) plot based on Bray–Curtis distance (Stress < 0.0001). (**G**) Relative abundance of dominant bacterial phyla in the CK and WX groups.

A total of 4245 operational taxonomic units (OTUs) were identified from all wheat rhizosphere soil samples by 16S rRNA gene sequencing. Of these, 1394 OTUs were shared between the oligosaccharide-coated group (WX) and the control group (CK) (Figure 9A). Alpha diversity analysis indicated that WX treatment did not significantly affect microbial richness or other α-diversity indices, as shown by Chao1 (Figure 9B), Shannon (Figure 9C), and Simpson (Figure 9D) indices (ns, no significant difference). However, Bray–Curtis-based PCoA (Figure 9E) and NMDS analyses (Stress < 0.0001, Figure 9F) revealed a significant shift in β-diversity, indicating that the microbial community structure was markedly altered and that specific taxa were selectively enriched under the WX treatment. Based on the observed overall community shifts, subsequent analyses focused on identifying key microbial taxa associated with the WX treatment and their potential roles in mediating seed germination and seedling growth.

At the phylum level, Proteobacteria, Bacteroidota, Firmicutes, Verrucomicrobiota and Actinobacteriota were identified as dominant taxa in the rhizosphere (relative abundance > 1%), as shown in the community composition bar plot (Figure 9G). The WX treatment significantly increased the relative abundance of Actinobacteriota, while significantly reducing Proteobacteria abundance (FDR < 0.05). In addition, Bacteroidota showed an increasing trend, whereas Firmicutes exhibited a decreasing trend (FDR < 0.1). These results indicated that the WX treatment substantially reshaped the phylum-level composition of the wheat rhizosphere microbiome.

**Figure 10:**
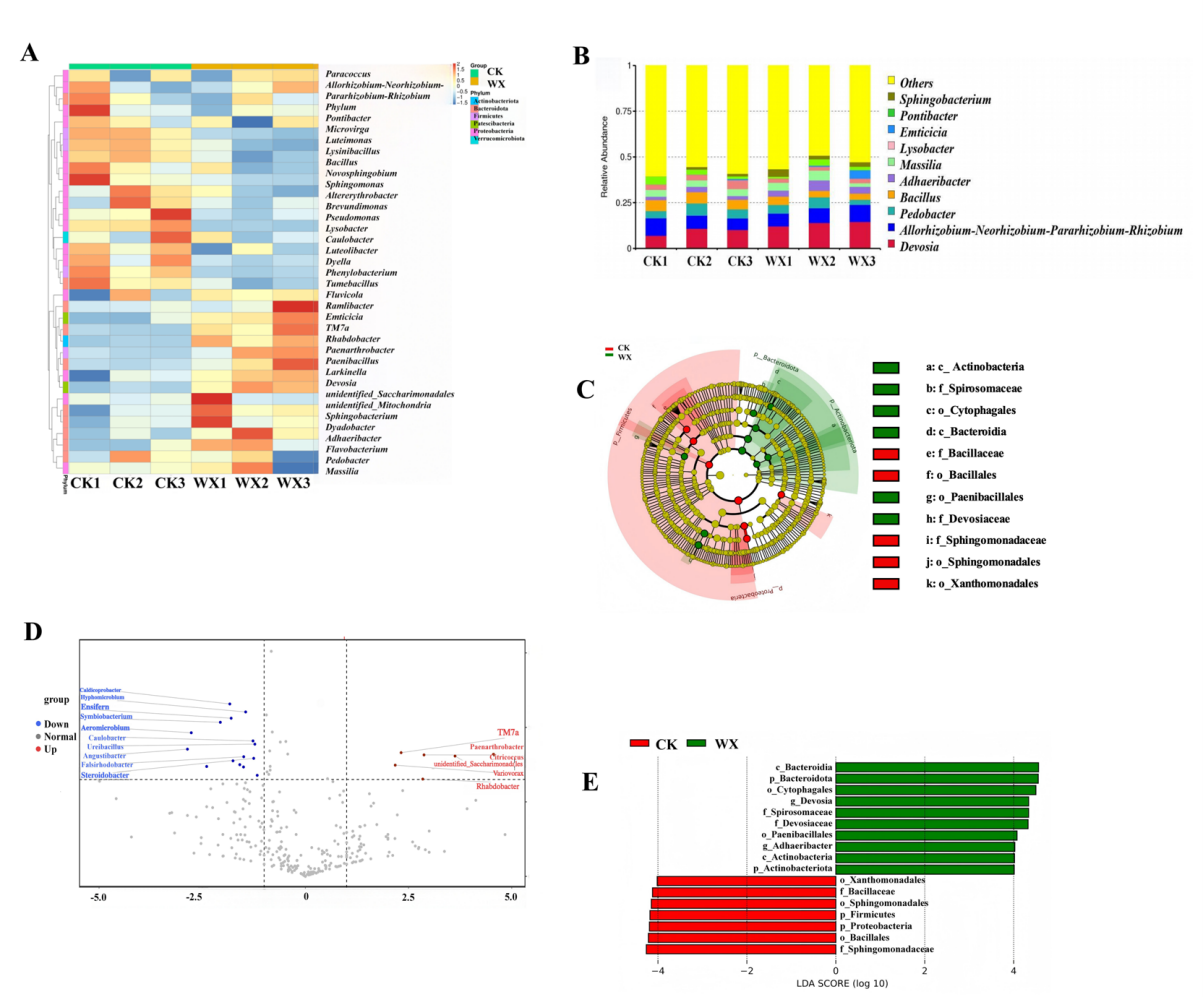
Genus-level composition and differential biomarkers of wheat rhizosphere bacterial communities under CK and WX treatments. (**A**) Heatmap showing the relative abundance profiles of the top 30 bacterial genera across samples. (**B**) Stacked bar plot showing the relative abundance of dominant bacterial genera at the genus level. (**C**) LEfSe cladogram showing phylogenetic distribution of biomarkers in the CK and WX groups. (**D**) Volcano plot showing significantly enriched (red) and depleted (blue) genera in the WX group compared with CK (|log_2_FC| > 1, *p* < 0.05). (**E**) LDA score plot of biomarkers identified by LEfSe analysis (LDA > 4.0).

At the genus level, pronounced shifts in community structure and functional potential were further observed (Figure 10A,B). WX treatment significantly enriched multiple genera associated with carbohydrate metabolism and organic matter decomposition, including unidentified_*Saccharimonadales*, *Rhabdobacter*, *Sphingobacterium*, *Flavobacterium*, *Dyadobacter* and *Larkinella*. In contrast, several functionally specialized groups were significantly suppressed, including methane-oxidizing bacteria (*Methylocaldum*, *Methylocella*), steroid-degrading bacteria (*Steroidobacter*), urease-associated *Ureibacillus*, nitrogen-fixing *Ensifer,* multiple strict anaerobes (e.g., *Dethiobacter, Caldicoprobacter*), and oligotrophic taxa such as *Hyphomicrobium* and *Caulobacter*. Notably, adhesion-associated bacteria (*Adhaeribacter*) and *TM7a* lineage taxa, which are often considered symbiotic or parasite-dependent microorganisms, were significantly enriched under the WX treatment (Figure 10C,E). These findings suggested that the WX treatment induced a more complex microbial restructuring process beyond simple nutrient competition, likely involving enhanced physical aggregation and intensified microbial interactions.

Furthermore, plant growth-promoting and bioremediation-related genera, including *Devosia*, *Variovorax* and *Paenarthrobacter*, as well as stress-resilient and biocontrol-associated taxa such as *Citricoccus* and *Ramlibacter*, were significantly enriched in the WX group. Among them, *Devosia* represented the most dominant genus across all treatments in the rhizosphere microbiome. In contrast, the CK samples retained higher relative abundances of conventional beneficial genera such as *Bacillus* and *Lysobacter*, while the abundance of *Devosia* was reduced. Volcano plot analysis (|log_2_FC| > 1, *p* < 0.05) further confirmed that *TM7a*, *Paenarthrobacter* and *Citricoccus* were among the most significantly enriched taxa in the WX group, whereas *Caldicoprobacter* and *Hyphomicrobium* were among the most significantly depleted taxa. In addition, LEfSe analysis (LDA > 4.0) supported these results at the phylogenetic level (Figure 10C,E). The WX-associated biomarkers were mainly distributed in Actinobacteriota (e.g., *Paenarthrobacter*, *Citricoccus*) and Bacteroidota (e.g., *Rhabdobacter*), as well as key Proteobacteria taxa such as *Devosia*. In contrast, the CK-associated microbial features were primarily defined by Firmicutes (e.g., *Bacillales)* and Proteobacteria (e.g., *Sphingomonadales*). Overall, the WX treatment acted as a high-quality carbon input that drove rhizosphere microbial succession (Figure 10A–E). A selective enrichment of fast-growing, organic matter-degrading r-strategists and specific beneficial taxa (e.g., *Devosia*, *Paenarthrobacter* and *Citricoccus*) was observed. This shift likely promoted wheat growth indirectly by enhancing soil nutrient mineralization and restructuring microbial interactions, rather than by simply enriching classical plant growth-promoting bacteria.

### 3.8. Integrated Microbiome–Transcriptome Analysis Revealed Potential Interactions Between Rhizosphere Microbiota and Plant Gene Expression

**Figure 11:**
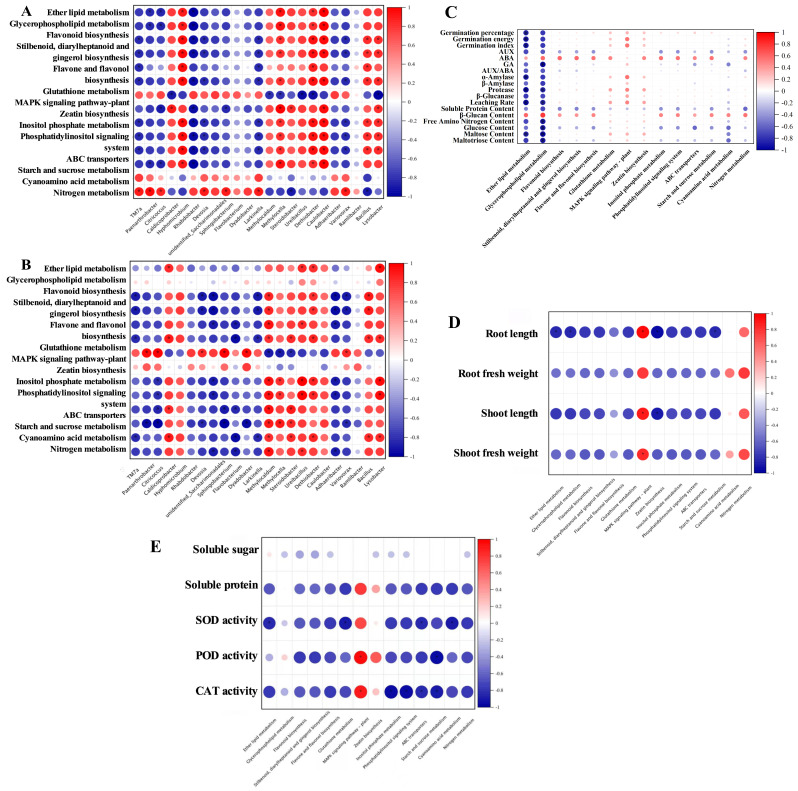
Correlation analysis between rhizosphere microbial genera, plant transcriptomic pathways, and physiological traits under WX treatment. (**A**,**B**) Heatmaps showing Spearman’s correlation coefficients between key root metabolic pathways and rhizosphere bacterial genera at the two-leaf stage (**A**) and late growth stage (**B**). (**C**–**E**) Heatmaps showing Spearman’s correlation coefficients between root metabolic pathways and physiological traits at the emergence stage (**C**), two-leaf stage (**D**), and late growth stage (**E**). Color scale represents correlation coefficient values (red, positive correlation; blue, negative correlation). Asterisks denote significant correlations (* *p* < 0.05).

To examine the dynamic associations between root gene expression and key physiological traits under the xanthan oligosaccharide treatment (WX), KEGG-enriched pathways and corresponding physiological parameters were subjected to correlation analysis across the emergence stage, two-leaf stage and late growth stage (Figure 11A–E). At the emergence stage, glycerophospholipid metabolism was found to be significantly negatively correlated with gibberellin content, free amino nitrogen and soluble sugars (glucose, maltose, and maltotriose), while a positive correlation was observed with β-glucan content (Figure 11C). Ether lipid metabolism was also significantly negatively correlated with germination rate, germination potential, germination index, leachate rate, and maltose content (Figure 11C). In contrast, the MAPK signaling pathway was positively correlated with germination potential and protease activity (Figure 11C). These results suggested that lipid metabolic pathways were suppressed, whereas MAPK signaling-related pathways were positively associated under the WX treatment, jointly promoting reserve mobilization and germination through hormonal rebalancing.

At the two-leaf stage, seedling growth became the dominant physiological process. Root growth was negatively correlated with glycerophospholipid metabolism, ether lipid metabolism, zeatin biosynthesis, and starch and sucrose metabolism, while positive correlations were observed with the MAPK signaling pathway (plant) and nitrogen metabolism (Figure 11E). In contrast, aboveground growth traits (seedling length and fresh weight) were consistently positively correlated with MAPK signaling but negatively correlated with zeatin biosynthesis (Figure 11E). These patterns indicated that root elongation and seedling biomass accumulation were associated with MAPK-mediated signaling and nitrogen assimilation support.

At the late growth stage, SOD activity was negatively correlated with glutathione metabolism, whereas POD and CAT activities were positively correlated with MAPK signaling and negatively correlated with carbon metabolism pathways (Figure 11D). These results suggested that, at later developmental stages, MAPK signaling-related pathways remained associated with antioxidant defense responses, while partial suppression of carbon metabolism may have facilitated resource reallocation toward reactive oxygen species (ROS) scavenging, thereby enhancing potential stress resilience.

### 3.9. Correlation Analysis Between Rhizosphere Microbial Community Shifts and Plant Transcriptomic Responses

To clarify how the xanthan oligosaccharide composite seed coating (WX) influenced wheat physiological responses via regulation of beneficial rhizosphere microbial communities, correlation analyses were performed between key root metabolic pathways and genus-level microbial abundance at the two-leaf stage (T stage) and late growth stage (L stage) (Figure 11A,B). At the rapid growth stage (T stage), metabolic pathways involved in membrane lipid biosynthesis were found to be negatively correlated with the abundances of putative beneficial taxa *Paenarthrobacter* and *Citricoccus*, as well as the organic matter-degrading genus *Rhabdobacter* (Figure 11A). In contrast, these pathways were positively correlated with oligotrophic taxa (*Hyphomicrobium*, *Caulobacter*), methylotrophic bacteria involved in one-carbon cycling (*Methylocella*), and strict anaerobes (*Dethiobacter*). Core antioxidant and defense-related pathways in roots, including flavonoid biosynthesis, stilbene, diarylheptanoid and gingerol biosynthesis, and glutathione metabolism, also showed significant positive correlations with oligotrophic taxa (*Hyphomicrobium*, *Caulobacter*), methylotrophs (*Methylocella*), strict anaerobes (*Dethiobacter*), as well as classical plant growth-promoting bacteria such as Bacillus and biocontrol-associated *Lysobacter* (Figure 11A). In contrast, these pathways were negatively correlated with *TM7a* lineage taxa, organic matter-degrading *Rhabdobacter*, *Devosia* and hormone-regulating *Variovorax*. Zeatin biosynthesis, which is closely associated with cell division and growth regulation, was negatively correlated with *Citricoccus* and *Rhabdobacter* but positively correlated with thermophilic bacteria (*Caldicoprobacter*) and methylotrophic taxa. Meanwhile, the MAPK signaling pathway, which is involved in stress perception and nutrient signaling, showed positive correlations with the core WX-enriched beneficial microbiota (*Paenarthrobacter, Citricoccus, Devosia, Sphingobacterium, Dyadobacter, Variovorax*) but negative correlations with stress-associated or suppressed taxa under the WX treatment, including *Caldicoprobacter*, *Methylocaldum*, *Methylocella*, *Dethiobacter* and biocontrol-associated *Lysobacter* (Figure 11A). In addition, inositol phosphate metabolism, ABC transporter pathways, and starch and sucrose metabolism were positively correlated with beneficial genera such as *Hyphomicrobium*, *Bacillus* and *Lysobacter* but negatively correlated with *TM7a* and *Devosia*. Nitrogen metabolism showed strong positive correlations with *Citricoccus*, *Devosia* and several organic matter-degrading taxa (*Sphingobacterium*, *Variovorax*), suggesting a potential role of these microbial groups in supporting early nitrogen acquisition in wheat.

At the late growth stage (L stage), the correlation pattern of starch and sucrose metabolism was markedly shifted (Figure 11B). This pathway was negatively correlated with the WX-enriched core beneficial microbiota, including *Paenarthrobacter*, *Citricoccus, Devosia*, *Sphingobacterium*, *Dyadobacter* and *Variovorax*, but positively correlated with functionally specialized taxa such as *Methylocaldum*, *Methylocella* and *Steroidobacter* (Figure 11B). These results suggested that carbon allocation strategies were shifted from vegetative growth support toward grain filling and nutrient remobilization. Beneficial taxa that were positively associated with carbon metabolism at the T stage (e.g., *Variovorax* and *Paenarthrobacter*) may have shifted toward competitive utilization of photosynthates at the L stage, whereas methylotrophic bacteria (*Methylocaldum*, *Methylocella*) became dominant microbial partners, potentially contributing to carbon remobilization and energy balance during senescence. At the same stage, cyanogenic amino acid metabolism showed a positive correlation with biocontrol-associated genera such as *Bacillus* and *Lysobacter*, further supporting enhanced defense requirements during late development. In contrast, negative correlations with genera such as *Flavobacterium* suggested a suppression of late-stage saprophytic communities that may otherwise weaken plant resistance. Furthermore, the microbial support network for nitrogen metabolism became more restricted at the L stage, with a preferential association with methylotrophic and ureolytic taxa. The genus *Ureibacillus*, which possesses urease activity, was suggested to contribute to nitrogen remobilization from protein degradation products in senescing tissues. The persistent positive correlation with *Methylocaldum* indicated that methylotrophic bacteria continued to play a key role in coupling carbon and nitrogen metabolism, likely contributing to C/N balance regulation during nutrient redistribution.

## 4. Discussion

The role of carbohydrate-based elicitors in plant immunity and growth regulation has been widely recognized. However, the mechanisms underlying their application as seed-coating agents remain insufficiently understood, particularly regarding how endogenous signaling networks and rhizosphere microbial communities are coordinately regulated to achieve simultaneous growth promotion and potential stress resilience. In the present study, an integrative analysis of wheat root transcriptomes, rhizosphere microbiomes, and physiological and biochemical traits was conducted. The results indicated that a xanthan gum oligosaccharide-based composite seed coating (WX) regulated seed germination, seedling growth, and potential stress resilience through coordinated modulation of endogenous hormone signaling, transcriptional reprogramming, and plant–microbe interactions. The regulatory effects of WX were consistently stronger than those of xanthan oligosaccharide (XOG) or wall material (WM) alone and were maintained from early seedling stages under controlled conditions to the whole growth period in the field. Unlike conventional single-target plant growth regulators, the WX coating established a multilayer regulatory network involving endogenous physiological modulation, molecular pathway reprogramming, and rhizosphere microbiome reconstruction, thereby enabling coordinated enhancement of growth and stress resilience.

Seed germination and early seedling establishment depend on efficient mobilization of endosperm reserves and rapid activation of root nutrient uptake and assimilation capacity [30]. During germination, starch, proteins, and cell wall polysaccharides stored in the endosperm are hydrolyzed to provide energy and structural precursors for embryo development. In this study, the WX treatment was found to rapidly activate the endosperm hydrolytic enzyme system, including α-amylase, β-amylase, protease and β-glucanase (Figure 2A–D). This activation promoted the release of soluble sugars and amino acids (Figure 4A–G), thereby establishing a metabolic foundation for germination vigor and seedling growth [31]. In parallel, enhanced root system development was promoted by the WX coating (Figure 1A–D), which provided a structural advantage for subsequent nutrient uptake and stress adaptation.

Transcriptomic analysis further revealed the upstream molecular mechanisms regulating hydrolytic activity and early seedling development. At the emergence stage, early responses were preferentially initiated by the WX coating, characterized by remodeling of membrane lipid metabolism and activation of transmembrane transport systems (Figure 7D and Figure 8A). Stress-related signaling was attenuated, as indicated by the downregulation of PLD2, and metabolic resources were preferentially allocated to growth processes. Meanwhile, carbon and nitrogen reserve mobilization was activated, as reflected by the upregulation of BGLU and NIA1 (Figure 8A), thereby providing initial energy for germination. These molecular changes were consistent with increased levels of soluble protein, polysaccharide-degrading enzyme activity, and carbon and nitrogen metabolites in the endosperm (Figure 2 and Figure 3). Notably, higher hydrolytic enzyme activities were observed in the WX-treated seeds than in the XOG and CK treatments, suggesting that the combination of wall material and oligosaccharide was associated with stronger endosperm reserve mobilization, including nitrogen-related mobilization, and may have contributed to improved germination.

In addition, root nutrient uptake and assimilation were closely associated with carbon and nitrogen metabolic capacity during later developmental stages [32]. At the two-leaf and one-heart stage, carbon catabolism was continuously enhanced, as evidenced by the upregulation of BGLU and BAM3, whereas sucrose synthesis and degradation pathways (SS2, CIN1/2/3) were broadly suppressed (Figure 7E and Figure 8B). In parallel, core nitrogen assimilation genes (NIA1, NRT2.1, GLNA2) were continuously upregulated, ensuring a sufficient supply and transport of soluble sugars and proteins. These changes supported rapid root elongation and increased shoot biomass, consistent with the observed growth phenotypes (Figure 1). At the late stage, carbon and nitrogen metabolic activities were attenuated (Figure 7F and Figure 8A), indicating a transition from rapid energy supply to metabolic homeostasis, thereby preventing excessive resource consumption. This pattern is consistent with previous reports showing that plants reprogram carbon and nitrogen fluxes under stimulation to balance growth and maintenance processes [33]. Furthermore, upregulation of GGT3 and UGT85B1 indicated that cyanogenic amino acid metabolism was integrated into secondary metabolism, providing additional amino acid precursors for growth. Collectively, the WX treatment established a continuous metabolic supply system through early enzymatic activation and staged reprogramming of carbon and nitrogen metabolism, ensuring seamless transition from reserve mobilization to root nutrient assimilation.

The promotion of seed germination and early seedling growth by the WX coating fundamentally depended on precise regulation of endogenous hormone homeostasis. Germination is governed by the balance between growth-promoting and growth-inhibiting hormones. In this study, the WX treatment significantly altered hormonal equilibrium by increasing auxin (IAA) and gibberellin (GA) levels while accelerating abscisic acid (ABA) degradation (Figure 4A–D). As a result, the IAA/ABA ratio was markedly increased during the critical germination window (48–72 h), indicating a shift from dormancy maintenance to growth initiation. This hormonal reprogramming provided a primary driving force for radicle protrusion and rapid seedling establishment. These observations are consistent with the regulatory patterns of oligosaccharide-based biostimulants in garden cosmos [2]. Importantly, the combined formulation with wall material enhanced the persistence and magnitude of hormonal responses, suggesting that the coating matrix improved oligosaccharide delivery efficiency.

At the molecular level, these physiological changes were associated with transcriptional regulation of cytokinin biosynthesis and stage-specific reprogramming of second messenger signaling pathways (Figure 8A–C). Cytokinin plays a central role in regulating cell division and differentiation and interacts with auxin and gibberellin signaling networks. In this study, cytokinin biosynthesis was continuously enriched from emergence to the two-leaf stage, as indicated by the upregulation of IPT5 and CYP735A2 and the downregulation of CKX4 and CISZOG1 (Figure 8B). This regulation promoted root meristem activity and provided a direct molecular basis for enhanced root growth. In parallel, phosphoinositide signaling was finely modulated. In the early stage, key kinase genes involved in inositol phosphate and phosphatidylinositol metabolism (ITPK4/5, IPK2) were suppressed, likely preventing excessive signal amplification and energy consumption. At the two-leaf stage, genes responsible for PIP2 formation (PIP5K9, PIP5K2) were specifically upregulated, while downstream signaling components were broadly downregulated (Figure 8A). This pattern suggested a strategy that maintained a stable membrane signaling platform while avoiding uncontrolled signal propagation. Consistent with this, PLD2 downregulation and LPP2 upregulation further indicated reduced stress signaling and improved membrane stability. In addition, ABC transporter genes related to auxin export (ABCB11) and ABA efflux (ABCG25) were continuously upregulated (Figure 8A–C), supporting directional hormone transport and reinforcing hormonal gradients required for growth regulation.

The enhancement of potential stress resilience by the WX coating was primarily attributed to the enrichment of the antioxidant defense system. Activities of SOD, POD, and CAT were significantly increased, with CAT showing the most pronounced response under the WX treatment (Figure 5A–C). This indicated that ROS detoxification capacity was strongly enhanced, contributing to the maintenance of redox homeostasis and the reduction in oxidative damage. Meanwhile, the accumulation of soluble sugars and proteins provided osmotic adjustment capacity and metabolic reserves for stress adaptation [34] (Figure 5D,E). Notably, chlorophyll content showed no significant difference among treatments (Figure 5F), indicating that potential stress resilience was achieved without compromising photosynthetic capacity, thereby avoiding additional metabolic costs. At the transcriptional level, a staged defense regulatory network was established (Figure 8A–C). During the early stages, glutathione metabolism genes (G6PD2, GSTX1) were upregulated, while MAPK signaling sensitivity and phenylpropanoid upstream pathways were suppressed, reducing unnecessary stress perception and conserving resources for growth. At the two-leaf stage, GST-mediated detoxification was selectively downregulated, whereas phenylpropanoid flux was redirected toward the synthesis of high-value antioxidants such as flavonoids and proanthocyanidins. At the late stage, CAT2 was upregulated, strengthening the H_2_O_2_ scavenging capacity, which was consistent with physiological CAT activity patterns (Figure 5B).

Rhizosphere microbiome shifts were observed in parallel with WX-induced physiological and transcriptomic changes, but these patterns should be interpreted as associations rather than direct evidence of functional interactions (Figure 11B–D). The WX coating was associated with increased relative abundances of taxa previously reported to have plant growth-promoting or organic matter-degrading potential, including *Devosia*, *Paenarthrobacter* and *Citricoccus*, while taxa related to methane oxidation, strict anaerobic metabolism and other specialized ecological functions showed lower relative abundances (Figure 11A–E). This shift suggested a possible change in community composition toward taxa with reported links to nutrient mineralization and stress-associated ecological functions, rather than demonstrating a direct functional transition of the rhizosphere microbiome. Organic matter degraders may be related to nutrient availability through their potential participation in soil organic matter turnover, thereby providing a plausible but unverified indirect association with plant growth [35]. Correlation analyses further indicated that these beneficial taxa were positively associated with nitrogen metabolism and MAPK signaling pathways (Figure 11A,B), suggesting co-variation between microbial community composition and host transcriptional responses rather than functional coupling between microbial activity and host stress responses. In contrast, suppressed microbial groups were positively associated with certain metabolic pathways, which may reflect niche redistribution and substrate-related microbial selection. At later stages, carbon metabolism correlations shifted, and methylotrophic bacteria became more prominent (Figure 11B), a pattern that may be associated with developmental changes in root exudation and carbon allocation, although direct measurements of exudates or microbial metabolic activity were not performed. These results support a correlation-based model in which rhizosphere microbial community shifts are associated with plant developmental status, nutrient-related pathways and stress-related transcriptional responses. However, causal relationships between enriched bacterial taxa and host gene expression remain to be validated through microbial isolation, re-inoculation assays, synthetic community experiments and gene-level functional analyses.

Although this study provides a comprehensive framework for understanding the WX-mediated regulation of wheat growth and potential stress resilience, several limitations remain. First, the analysis focused mainly on germination and seedling stages, and long-term effects on yield formation under multi-year field conditions require further validation. Second, the key genes and microbial taxa identified by omics analyses were not experimentally validated. Future studies should employ gene editing, microbial isolation, and re-inoculation experiments to confirm their functional roles.

In conclusion, this study indicates that the WX coating is associated with improved wheat growth and stress-related physiological responses, accompanied by changes in hormone profiles, staged transcriptional reprogramming, and shifts in rhizosphere microbial community composition. Rather than establishing a direct causal regulatory network, the observed associations among hormonal homeostasis, root transcriptomic changes, and microbiome restructuring support a correlation-based multilayer model that may explain the biological effects of WX treatment. These findings expand the theoretical understanding of oligosaccharide-based biostimulants and provide a scientific basis for the development of sustainable and efficient agricultural formulations. The WX seed coating is simple to prepare and shows growth-promoting performance under the tested conditions, with promising potential for application in wheat production systems. However, its effects on yield formation and stress resilience require further validation through multi-year field trials, defined stress-challenge experiments, microbial isolation, re-inoculation assays and functional verification of key host genes and bacterial taxa.

## Figures and Tables

**Table 1:** Endosperm leaching index of wheat seeds at 72 h post-germination.

Endosperm Dissolution Index	CK	WM	XOG	WX
Leaching rate (%)	74.5 ±4.2 b	82.5 ± 3.3 ab	86.6 ± 5.9 a	88.7 ± 5.0 a
Soluble protein content (%)	4.3 ± 0.2 c	4.9 ± 0.5 c	6.4 ± 0.3 b	9.6 ± 0.1 a
β-glucan content (mg/L)	133.4 ± 15.6 a	121.3 ± 10.8 ab	107.8 ± 12.3 b	56.9 ± 7.2 c
Free amino nitrogen content (mg/100 g)	152.8 ± 5.4 c	179.3 ± 4.4 b	183.3 ± 6.4 b	220.7 ± 6.7 a
Glucose content (g/L)	10.1 ± 0.4 c	13.3 ± 2.3 c	20.9 ± 1.4 b	28.4 ± 0.5 a
Maltose content (g/L)	50.3 ± 1.2 d	62.7 ± 2.1 c	89.3 ± 3.1 b	120.2 ± 1.5 a
Maltotriose content (g/L)	8.1 ± 1.2 c	9.5 ± 0.8 c	11.6 ± 0.6 b	14.7 ± 0.3 a

Note: Values are presented as mean ± standard deviation from three technical replicates. For each treatment and sampling time point, multiple wheat seed samples were pooled and homogenized, and the homogenized sample was divided into three aliquots for independent measurement. Therefore, the reported variation represents analytical variation among technical replicates rather than variation among independent biological replicates. The different lowercase letters within the same row indicate significant differences among treatments at *p* < 0.05.

## Data Availability

The original contributions presented in this study are included in the article. Further inquiries can be directed to the corresponding author.
